# Macrophage Mitochondrial Biogenesis and Metabolic Reprogramming Induced by Leishmania donovani Require Lipophosphoglycan and Type I Interferon Signaling

**DOI:** 10.1128/mbio.02578-22

**Published:** 2022-10-12

**Authors:** Hamlet Acevedo Ospina, Marie-Michèle Guay-Vincent, Albert Descoteaux

**Affiliations:** a INRS–Centre Armand-Frappier Santé Biotechnologie, Laval, Quebec, Canada; Harvard T. H. Chan School of Public Health

**Keywords:** IFNAR, *Leishmania*, lipophosphoglycan, macrophages, mitochondria

## Abstract

Pathogen-specific rewiring of host cell metabolism creates the metabolically adapted microenvironment required for pathogen replication. Here, we investigated the mechanisms governing the modulation of macrophage mitochondrial properties by the vacuolar pathogen *Leishmania*. We report that induction of oxidative phosphorylation and mitochondrial biogenesis by Leishmania donovani requires the virulence glycolipid lipophosphoglycan, which stimulates the expression of key transcriptional regulators and structural genes associated with the electron transport chain. *Leishmania*-induced mitochondriogenesis also requires a lipophosphoglycan-independent pathway involving type I interferon (IFN) receptor signaling. The observation that pharmacological induction of mitochondrial biogenesis enables an avirulent lipophosphoglycan-defective L. donovani mutant to survive in macrophages supports the notion that mitochondrial biogenesis contributes to the creation of a metabolically adapted environment propitious to the colonization of host cells by the parasite. This study provides novel insight into the complex mechanism by which *Leishmania* metacyclic promastigotes alter host cell mitochondrial biogenesis and metabolism during the colonization process.

## INTRODUCTION

*Leishmania* is a trypanosomatid parasite responsible for a spectrum of human diseases called leishmaniasis (1). This parasite is transmitted to mammals by phlebotomine sand flies through the inoculation of metacyclic promastigotes, which are internalized by phagocytic cells (2). There, they create specialized parasitophorous vacuoles that support their differentiation and replication as amastigotes (3) Given their auxotrophies for several essential metabolites they must acquire from their host (4–6), *Leishmania* parasites rewire or alter diverse host cell metabolic pathways during the infection process (6, 7). In particular, a growing number of studies indicate that *Leishmania* targets host cell energy metabolism to create an environment permissive to their replication. Hence, studies on the transcriptional signature of macrophages infected with L. major suggested an enhanced rate of glycolysis and lactate production with reduced pyruvate flux through the tricarboxylic acid cycle, indicating that infected macrophages would tend to convert glucose into lactate even in the presence of sufficient oxygen to support mitochondrial oxidative phosphorylation (OXPHOS) (8). Bioenergetics profiling of macrophages infected with various *Leishmania* species revealed an induction of glycolysis during the early phases of infection, followed by a switch from glycolysis to OXPHOS during the late phase of infection (9, 10). Analysis of the molecular mechanisms by which L. infantum modulates mitochondrial metabolism revealed an essential role for the metabolic sensor AMP-activated protein kinase (AMPK) (9), a key modulator of peroxisome proliferator-activated receptor gamma (PPARγ) coactivator 1α (PGC-1α) activity (11, 12). PGC-1α is a transcriptional coactivator and is the master switch that integrates mitochondrial biogenesis and energy-generating functions of mitochondria with metabolic demands associated with physiological states associated with health and disease (13–16). Hence, PGC-1α controls multiple aspects of mitochondrial biogenesis, including increased mitochondrial number and biogenesis of the OXPHOS system (13), by coordinating the concerted expression of nuclear and mitochondrial genes encoding proteins involved in these processes (16–18).

Macrophages and other phagocytes are highly plastic cells that rapidly adapt their metabolism in response to invading pathogens. It is well documented that the interplay between pathogen-derived molecules and host innate immune receptors contributes significantly to these metabolic changes (19, 20). Hence, exposure of monocytes to bacterial lipopolysaccharide (LPS), which stimulates Toll-like receptor 4 (TLR4), induces an increase in glycolysis and a decrease in OXPHOS, whereas the TLR2 ligand Pam_3_CysSK_4_ (a synthetic lipopeptide) upregulates both glycolysis and OXPHOS (21). However, studies with live intracellular pathogens revealed a diversity of macrophage metabolic changes consistent with the notion of host-driven and pathogen-driven metabolic rewiring (22). This notion reflects the fact that pathogens produce several effectors and virulence factors that dynamically interact with various host cell innate immune receptors and molecules over the course of infection. Of interest, Mycobacterium tuberculosis decreases both glycolysis and OXPHOS in infected macrophages, and recent evidence indicates that type I interferon (IFN) expressed by these cells plays a central role in decreasing macrophage energy metabolism during mycobacterial infection (23, 24).

Whereas the role of the sirtuin 1 (SIRT1)-AMP-activated protein kinase (AMPK) axis in mediating *Leishmania*-induced changes in the host cell bioenergetics profile has been established (9), our knowledge of the nature of both the host cell receptors and *Leishmania* effectors involved in the macrophage metabolic reprogramming remains fragmentary. In the present study, we investigated the mechanisms by which L. donovani metacyclic promastigotes alter host cell mitochondrial biology. In particular, we sought to determine the potential role of the parasite cell surface glycolipid lipophosphoglycan (LPG) in this process (25). This virulence factor contributes to the ability of *Leishmania* promastigotes to successfully colonize phagocytic cells and to alter the innate immune response by downmodulating macrophage microbicidal functions, inducing the secretion of cytokines, and shaping the parasitophorous vacuole in which *Leishmania* replicates (25–29). We report that L. donovani metacyclic promastigotes induce at least two distinct host cell responses in the context of the modulation of macrophage mitochondrial biogenesis, which are mediated by TLR4 and endosomal TLRs. One is the LPG-dependent enhancement of macrophage mitochondrial mass, increased expression of PGC-1α and of genes associated with the electron transport chain, and stimulation of OXPHOS. The other response is the LPG-independent induction of alpha interferon (IFN-α) expression, which also mediates enhancement of macrophage mitochondrial mass but has no impact on the induction PGC-1α expression or on mitochondrial flux.

## RESULTS

### LPG is required for the alteration of host cell bioenergetic metabolism by L. donovani metacyclic promastigotes.

**FIG 1:**
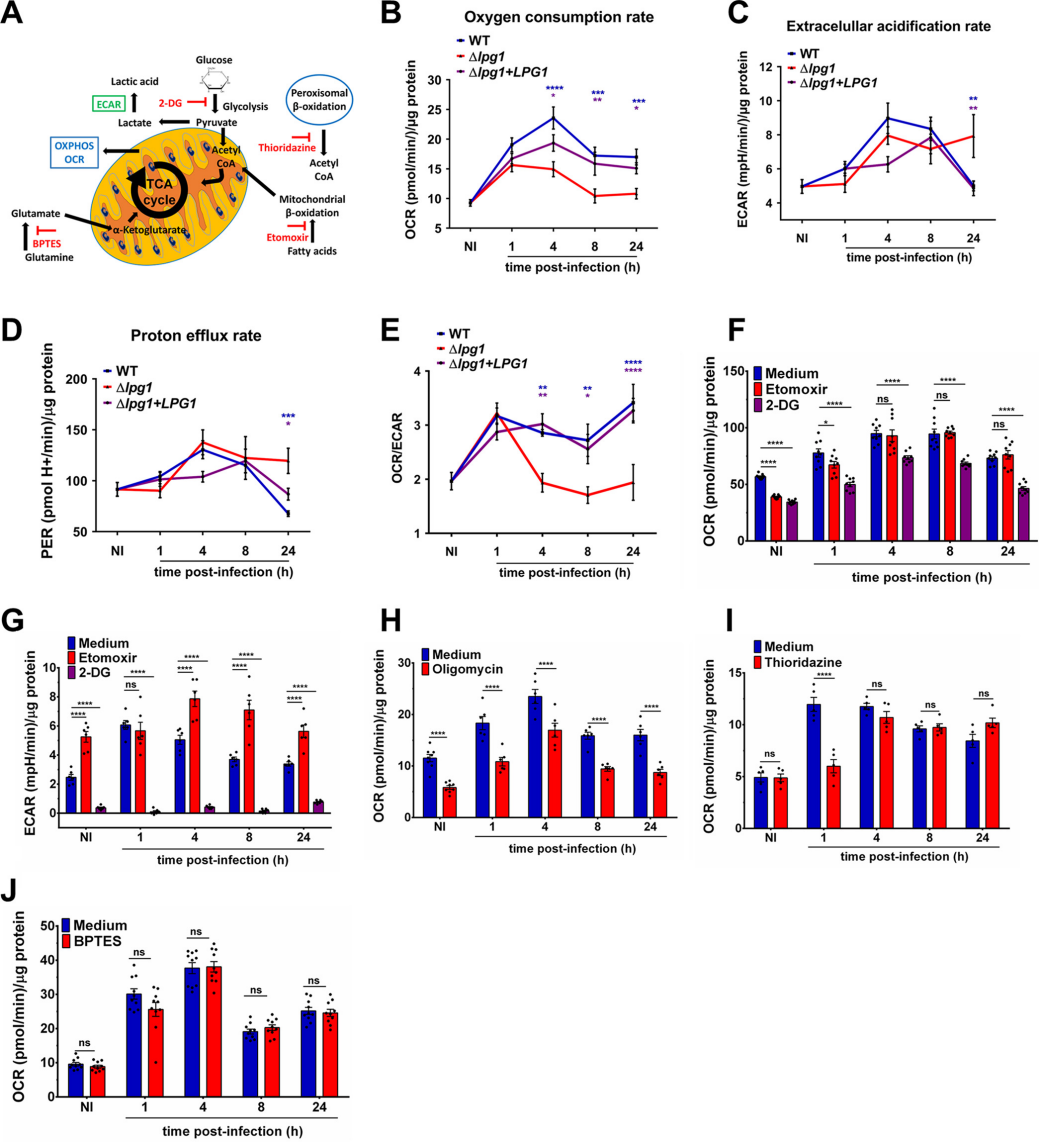
LPG contributes to the alteration of host cell bioenergetic metabolism by L. donovani metacyclic promastigotes. (A) Schematic showing the metabolic pathways detailing readouts for OCR, ECAR, and the carbon dependence source using inhibitors for glycolysis, mitochondrial β-oxidation, glutaminolysis, and peroxisomal β-oxidation. (B, C, D, and E) BMM were infected with either WT, Δ*lpg1*, or Δ*lpg1*+*LPG1* L. donovani metacyclic promastigotes, and at the indicated time points OCR, ECAR, PER, and the OCR/ECAR ratio were determined. (F and G) BMM were infected with WT L. donovani metacyclic promastigotes, and 30 min prior to the readouts for OCR and ECAR, 50 mM 2 deoxy-d-glucose (2-DG) or 4 μm etomoxir was added to the cells. (H, I, and J) BMM were infected with WT L. donovani metacyclic promastigotes, and 30 min prior to the readouts for OCR, 1 μM oligomycin, 1 μM thioridazine, or 3 μM BPTES was added to the cells. The readouts in each sample were normalized using the protein concentration, and the measurements were expressed as (OCR, ECAR, and PER)/μg protein. Representative graphic for three independent experiments (F to J). The data are presented as mean values ± SEM from three independent experiments. ****, *P* < 0.0001; ***, *P* < 0.001; **, *P* < 0.01; *, *P* < 0.05 according to a two-way ANOVA with Dunnett’s multiple-comparation test.

Whereas previous studies revealed that *Leishmania* promastigotes modulate host macrophage metabolism (8–10, 30), little is known regarding the parasite molecules involved in this process. We therefore sought to determine the potential role of the abundant cell surface virulence glycolipid LPG on the dynamics of host cell mitochondrial function induced by L. donovani metacyclic promastigotes. First, we assessed the impact of this glycolipid on the bioenergetic profile of bone marrow-derived macrophages (BMM) infected with either wild-type (WT) L. donovani metacyclic promastigotes, an isogenic LPG-defective mutant (Δ*lpg1*), or its complemented counterpart (Δ*lpg1*+*LPG1*) (see Fig. S1A in the supplemental material). We used live cell extracellular flux analysis to determine the oxidative metabolism of infected macrophages by measuring the mitochondrial oxygen consumption rate (OCR) as well as glycolysis through the measurement of the extracellular acidification rate (ECAR) (Fig. 1A). In BMM infected with either WT or Δ*lpg1*+*LPG1* L. donovani metacyclic promastigotes, we observed an increase in OCR values which peaked at 4 h postinfection and partially declined by 24 h (Fig. 1B). In contrast, in BMM infected with the Δ*lpg1* mutant, OCR remained significantly lower at all time points postinfection, suggesting that LPG is required to stimulate the oxidative metabolism of infected macrophages. For glycolysis, in BMM infected with WT, Δ*lpg1*, and *Δlpg1+LPG1* metacyclic promastigotes, we observed an increased in ECAR values compared to noninfected macrophages at 4 h and 8 h postphagocytosis, corresponding to an increase in the glycolytic flux (Fig. 1C). At 24 h postinfection, whereas the ECAR levels returned to the levels observed in noninfected cells for BMM infected with either WT or Δ*lpg1*+*LPG1* parasites, it remained elevated in BMM infected with Δ*lpg1* metacyclic promastigotes (Fig. 1C). The increase in ECAR was associated with an increased proton efflux rate in infected macrophages, which was significantly higher in BMM infected with Δ*lpg1* promastigotes than in BMM infected with either WT or Δ*lpg1*+*LPG1* promastigotes at 24 h postphagocytosis. (Fig. 1D). These results suggested that L. donovani metacyclic promastigotes induce an increase in glycolysis during the early phases of infection, which remains elevated in the absence of LPG. The elevated OCR/ECAR ratio observed for BMM infected with either WT or Δ*lpg1*+*LPG1* parasites indicates that higher OXPHOS takes place in these cells than in BMM infected with Δ*lpg1* parasites (Fig. 1E). To rule out the possibility that the increase in the OCR/ECAR ratio induced by L. donovani metacyclic promastigotes was the consequence of a phagocytic stimulus, we measured the OCR/ECAR ratio in BMM fed zymosan. As shown in Fig. S1B, phagocytosis of zymosan caused an important reduction in the basal OCR/ECAR ratio at 1 h, 4 h, 8 h, and 24 h postphagocytosis, indicating a reduction in the oxidative metabolism of BMM. Treatment with LPS induced a similar decrease in the OCR/ECAR ratio (Fig. S1C), consistent with increased glycolysis and reduced OXPHOS (21, 31). Additional control experiments confirmed that free L. donovani metacyclic promastigotes by themselves do not contribute to the OCR and ECAR measurements in infected BMM (Fig. S1D and E). To further characterize the process by which L. donovani metacyclic promastigotes modulate mitochondrial metabolic flux in BMM, we evaluated additional parameters of mitochondrial function. Hence, we observed an LPG-dependent increase in basal respiration, mitochondrial ATP production, nonmitochondrial oxygen consumption, and proton leak (Fig. S2A to E). In contrast, the observed increase in the spare respiratory capacity, which was calculated by the difference between the maximal OCR determined in the presence of the uncoupler fluoro-carbonyl cyanide phenylhydrazone (FCCP), was independent of LPG (Fig. S2F). Collectively, these results indicate that L. donovani metacyclic promastigotes induce an increase in both glycolysis and OXPHOS in infected BMM. Whereas LPG has little role to play in the increase in glycolysis, it contributes to OXPHOS in infected macrophages. Having shown that L. donovani metacyclic promastigotes modulate host cell metabolism, we next sought to identify the carbon dependence source to conduct energy production in infected BMM (Fig. 1A). Inhibition of the glycolytic pathway with 2 deoxy-d-glucose (2-DG) 30 min prior to the readouts significantly reduced L. donovani-induced OCR and ECAR (Fig. 1F and G). On the other hand, while inhibition of mitochondrial β-oxidation with etomoxir 30 min prior to the readouts had no major impact on OCR (Fig. 1F), we observed a significant increase in ECAR at 4 h, 8 h, and 24 h postphagocytosis (Fig. 1G). These results indicate that L. donovani metacyclic promastigotes promote an increase in host cell mitochondrial activity in a glycolytic-dependent manner and that blocking mitochondrial β-oxidation increases extracellular acidification. The reduction of OCR upon inhibition of the glycolytic pathway can partially explain where energy comes from (20% to 40%). Thus, we next sought to identify other carbon sources. Inhibition of ATP synthase (complex V) with oligomycin 30 min prior to the readouts significantly reduced L. donovani-induced OCR at 1 h, 4 h, 8 h, and 24 h postphagocytosis (Fig. 1H). Inhibition of peroxisomal β-oxidation with thioridazine 30 min prior to the readouts significantly reduced L. donovani-induced OCR at 1 h postphagocytosis and had no major impact on OCR at 4 h, 8 h, and 24 h postphagocytosis (Fig. 1I). On the other hand, inhibition of the glutamine oxidation pathway with BPTES [bis-2-(5-phenylacetamido-1,3,4-thiadiazol-2-yl)ethyl sulfide] 30 min prior to the readouts, had no major impact on OCR in L. donovani-infected BMM (Fig. 1J). These results indicate that increased OCR in L. donovani-infected macrophages is independent of glutaminolysis, is partially supported by peroxisomal β-oxidation during the first hour postphagocytosis, and is complex V-dependent.

10.1128/mbio.02578-22.1 FIG S1 (A) LPG levels from L. donovani, Δ*lpg1*, and Δ*lpg1*+*LPG1* were determined by Western blot analysis. Aldolase was used as a loading control. (B) BMM were stimulated with zymosan and at the indicated time points, and the OCR/ECAR ratio was analyzed. (C) BMM were stimulated with 100 ng/mL LPS, and at the indicated time points, the OCR/ECAR ratio was analyzed. (D and E) OCR (D) and ECAR (E) measurements were made using free L. donovani metacyclic promastigotes using the same doses as in Fig. 1. Basal OCR of noninfected BMM was used as a control. The data are presented as mean values ± the standard error of the mean (SEM) from three independent experiments using the most representative in each experiment. (B and C) ****, *P < *0.0001 according to a one-way ANOVA with Dunnett’s multiple-comparison test. (D and E) ****, *P < *0.0001 according to an unpaired *t* test with Welch's correction. Download FIG S1, TIF file, 0.9 MB. Copyright © 2022 Acevedo Ospina et al. 2022 Acevedo Ospina et al. https://creativecommons.org/licenses/by/4.0/ This content is distributed under the terms of the Creative Commons Attribution 4.0 International license.

10.1128/mbio.02578-22.2 FIG S2 (A) Schematic showing the different mitochondrial parameters evaluated. (B to F) BMM were infected with either WT, Δ*lpg1*, or Δ*lpg1*+*LPG1* L. donovani metacyclic promastigotes, and at the indicated time points, the (B) basal respiration, (C) mitochondrial ATP production, (D) nonmitochondrial oxygen consumption, (E) proton leak, and (F) spare respiratory capacity were determined. The readouts in each sample were normalized using the protein concentration, and the measurements were expressed as OCR/μg protein. The data are presented as mean values ± SEM from three independent experiments. ****, *P* < 0.0001; ***, *P* < 0.001; **, *P* < 0.01; *, *P* < 0.05 according to a two-way ANOVA with Dunnett’s multiple-comparison test. Download FIG S2, TIF file, 1.3 MB. Copyright © 2022 Acevedo Ospina et al. 2022 Acevedo Ospina et al. https://creativecommons.org/licenses/by/4.0/ This content is distributed under the terms of the Creative Commons Attribution 4.0 International license.

### L. donovani metacyclic promastigotes stimulate host cell mitochondrial biogenesis in an LPG-dependent manner.

**FIG 2:**
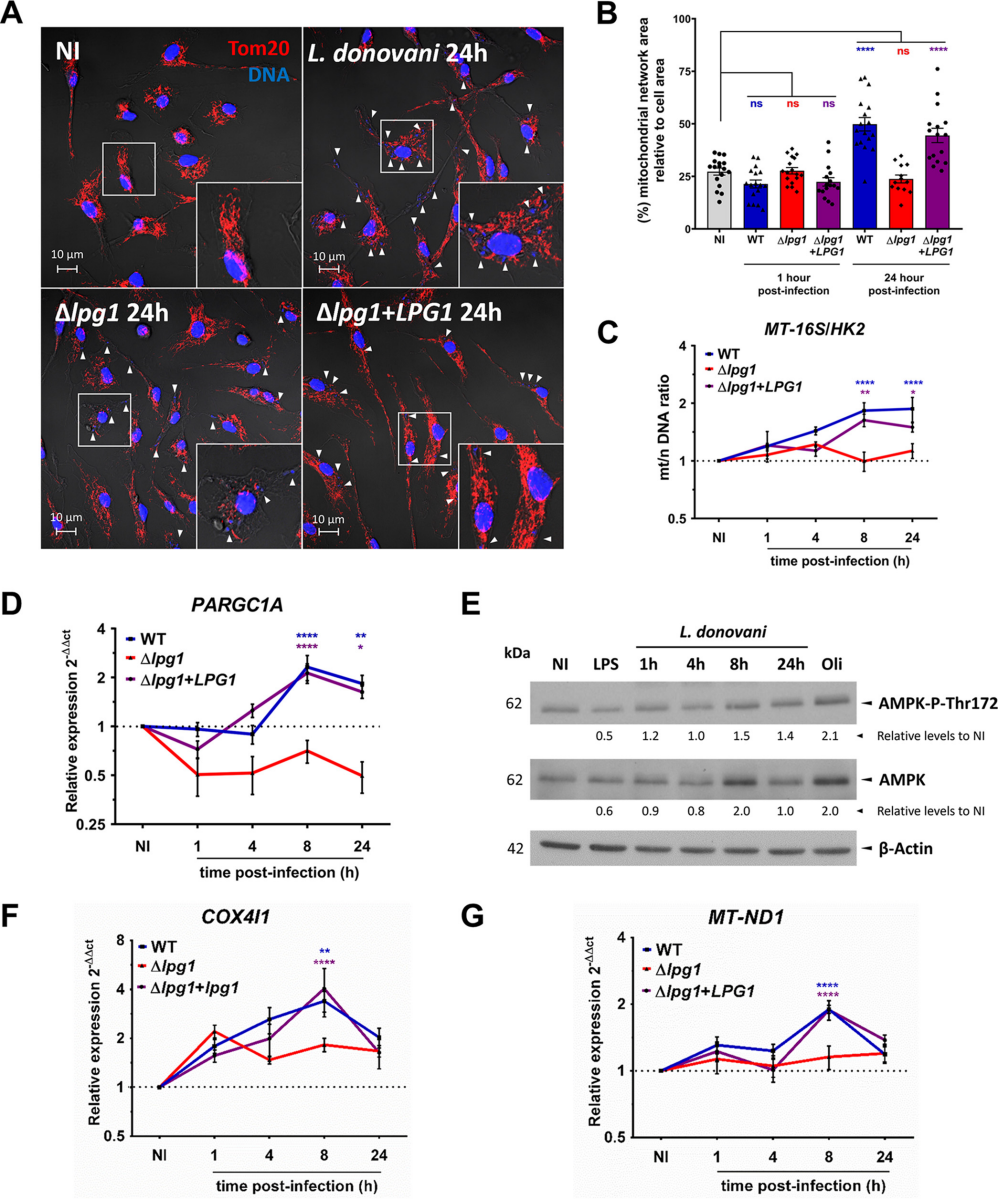
L. donovani metacyclic promastigotes stimulate host cell mitochondrial biogenesis in an LPG-dependent manner. (A) Distribution of Tom20 (red) in BMM infected for 24 h with either WT, Δ*lpg1*, or Δ*lpg1*+*LPG1* L. donovani metacyclic promastigotes. DAPI staining for DNA is in blue, and 2×-enlarged insets of representative mitochondria regions are shown. (B) Quantification of the mitochondrial network area relative to the cell area. (C) Mitochondrial/nuclear (mt/n) DNA ratio (*MT-16S/HK2*) in BMM infected with either WT, Δ*lpg1*, or Δ*lpg1*+*LPG1* L. donovani metacyclic promastigotes. (D) Kinetics of *PARGC1A* expression in BMM infected with either WT, Δ*lpg1*, or Δ*lpg1*+*LPG1* L. donovani metacyclic promastigotes. (E) Protein levels of AMPK-P-Thr172 and AMPK in BMM infected with L. donovani metacyclic promastigotes. BMM treated with 2.5 μM oligomycin for 30 min were used as the positive control for AMPK-P-Thr172. β-Actin was used as a loading control. Representative immunoblots for two independent experiments. (F and G) Kinetics of (F) *COX4I1* and (G) *MT-ND1* expression in BMM infected with either WT, Δ*lpg1*, or Δ*lpg1*+*LPG1* L. donovani metacyclic promastigotes. The data are presented as means values ± SEM from three independent experiments. ****, *P* < 0.0001; **, *P* < 0.01; *, *P* < 0.05 according to a one-way ANOVA with Dunnett’s multiple-comparation test.

To further investigate the contribution of LPG to the metabolic changes induced in macrophages by L. donovani promastigotes, we assessed the impact of this glycolipid on host cell mitochondrial biogenesis. To this end, we used immunofluorescence confocal microscopy to quantify alterations in the mitochondrial network area labeled with the outer mitochondrial membrane receptor Tom20. Compared to uninfected BMM, we observed a doubling in the mitochondrial network area relative to the cell area in BMM infected with either WT or Δ*lpg1*+*LPG1* L. donovani metacyclic promastigotes for 24 h (Fig. 2A and B and Fig. S3A). In contrast, the mitochondrial network area remained unaltered in BMM infected with Δ*lpg1* metacyclic promastigotes (Fig. 2A and B and Fig. S3A), suggesting that LPG participates in mitochondrial biogenesis induction in BMM infected with L. donovani promastigotes. To further demonstrate the impact of LPG on mitochondrial biogenesis, we infected BMM with either WT, Δ*lpg1*, or Δ*lpg1*+*LPG1* L. donovani metacyclic promastigotes, and we measured the mitochondrial/nuclear (mt/n) DNA ratio by quantitative PCR (qPCR), using two mitochondrion-encoded genes, 16S ribosomal (*MT-16S*) and NADH dehydrogenase 1 (*MT-ND1*) and the nuclear-encoded gene hexokinase-2 (*HK2*) for normalization. As shown in Fig. 2C and Fig. S3B, WT and Δ*lpg1*+*LPG1* metacyclic promastigotes induced a 2-fold increase in the ratio of *MT-16S* and *MT-ND1* relative to *HK2* (mt/n DNA ratio) at 8 h and 24 h postinfection compared to uninfected cells. In contrast, the mt/n DNA ratio remained unaltered in BMM infected with Δ*lpg1* metacyclic promastigotes (Fig. 2C and Fig. S3B), consistent with a requirement for LPG in the induction. An increase in the mt/n DNA ratio was induced at a low parasite-to-macrophage ratio and was not significantly augmented at higher parasite loads (Fig. S3C). This macrophage response was also induced by metacyclic promastigotes from other *Leishmania* species, and the extent and kinetics of increased mitochondrial DNA (mtDNA) content in BMM infected with L. mexicana, L. major, and L. amazonensis metacyclic promastigotes were similar to those observed in BMM infected with L. donovani (Fig. S3D).

10.1128/mbio.02578-22.3 FIG S3 (A) Distribution of Tom20 (red) in BMM infected for 1 h with either WT, Δ*lpg1*, or Δ*lpg1*+*LPG1* L. donovani metacyclic promastigotes. DAPI (4′,6-diamidino-2-phenylindole) staining for DNA is in blue, and 2×-enlarged insets of representative mitochondrial regions are shown. (B) Mitochondrial/nuclear (mt/n) DNA ratio (*MT-ND1/HK2*) in BMM infected with either WT, Δ*lpg1*, or Δ*lpg1*+*LPG1* L. donovani metacyclic promastigotes. (C) Mitochondrial/nuclear (mt/n) DNA ratio (*MT-16S/HK2*) in BMM infected with increasing MOIs of WT L. donovani metacyclic promastigotes. (D) Mitochondrial/nuclear (mt/n) DNA ratio (*MT-16S/HK2*) in BMM infected with L. donovani, L. mexicana, L. major, and L. amazonensis metacyclic promastigotes. (E to H) Kinetics of (E) *NRF1*, (F) *NDUFA9*, (G) *MT-CO1*, and (H) *MT-CO2* expression in BMM infected with either WT, Δ*lpg1*, or Δ*lpg1*+*LPG1* L. donovani metacyclic promastigotes. (I) Protein levels of NDUFA9, COX IV, and Tom20 in BMM infected with L. donovani. (J) Protein levels of AMPK-P-Thr172 and AMPK in BMM infected with either WT or Δ*lpg1* metacyclic promastigotes. BMM were treated with 0.1 mM AICAR for 4 h as a positive control for AMPK-P-Thr172. β-Actin was used as a loading control. Representative immunoblots for two independent experiments. The data are presented as mean values ± SEM from three independent experiments. (C and I) ****, *P* < 0.0001; **, *P* < 0.01; *, *P* < 0.05 according to a two-way ANOVA with Dunnett’s multiple-comparison test. Download FIG S3, TIF file, 1.7 MB. Copyright © 2022 Acevedo Ospina et al. 2022 Acevedo Ospina et al. https://creativecommons.org/licenses/by/4.0/ This content is distributed under the terms of the Creative Commons Attribution 4.0 International license.

### L. donovani metacyclic promastigotes induce the expression of genes associated with mitochondrial biogenesis in an LPG-dependent manner.

Mitochondrial biogenesis is a highly coordinated process that requires the expression of nuclear and mitochondrial genes and is regulated by specific signaling modules, transcription factors, and regulators of gene expression (14, 15, 32). We evaluated the impact of L. donovani metacyclic promastigotes and of LPG on the expression kinetics of the genes encoding PGC-1α (*PARGC1A*) and nuclear respiratory factor 1 (*NRF1*), two key regulators of gene expression associated with mitochondrial biogenesis and metabolism (14, 15, 32). We included in our analysis the phosphorylation of AMPK, a kinase which participates in mitochondrial biogenesis by potentiating the transcriptional activity of PGC-1α (11, 14). We observed that both WT and Δ*lpg1*+*LPG1* metacyclic promastigotes induced a 2-fold increase in the expression of *PARGC1A* at 8 h and 24 h postinfection and a 1.5-fold increase in the expression of *NRF1* at 8 h postinfection. In contrast, neither *PARGC1A* nor *NRF1* were induced by the Δ*lpg1* mutant (Fig. 2D, Fig. S3E). Interestingly, L. donovani metacyclic promastigotes, but not LPS, activated the phosphorylation of AMPK on Thr172, independently of LPG (Fig. 2E, Fig. S3J). We next assessed the expression of genes encoding components of the electron transport chain and associated with mitochondrial metabolism in infected BMM. We observed that both WT and Δ*lpg1*+*LPG1* metacyclic promastigotes increased by 2- to 3-fold the expression of genes encoded in the host cell nuclear (*COX4I1* and *NDUFA9*) and mitochondrial (*MT-ND1*, *MT-CO1*, *MT-CO2*) genomes (Fig. 2F and G, Fig. S3F to H). In contrast, expression of those genes remained unchanged in BMM infected with Δ*lpg1* metacyclic promastigotes (Fig. 2F and G, Fig. S3F to H). Western blot analyses confirmed the increased levels of mitochondrial proteins COX IV, NDUFA9, and Tom20 in BMM infected for 24 h with WT L. donovani metacyclic promastigotes compared to unstimulated BMM (Fig. S3I). These results are consistent with a requirement for LPG in the induction of mitochondrial biogenesis by L. donovani promastigotes. We next determined whether LPG is sufficient to stimulate mitochondrial biogenesis. To this end, we incubated BMM with either heat-killed or live L. donovani metacyclic promastigotes for 8 h, and we included polystyrene beads as phagocytic controls. As shown in Fig. S4A and B, only live WT L. donovani stimulated an increased mt/n (*MT-16S/HK2*) DNA ratio and *PARGC1A* gene expression. Additionally, we fed BMM with either zymosan or LPG-coated zymosan and measured the mt/n (*MT-16S/HK2*) DNA ratio and the expression of *PARGC1A* at 8 h postinternalization. Similar to WT L. donovani promastigotes, phagocytosis of LPG-coated zymosan resulted in the rapid redistribution of LPG within BMM (Fig. S4C). However, as shown in Fig. S4D and E, neither zymosan nor LPG-coated zymosan stimulated mitochondrial biogenesis as assessed by measuring the mt/n (*MT-16S/HK2*) DNA ratio and the expression of *PARGC1A* at 8 h postinternalization. To rule out the possibility that serum-opsonization influenced the stimulation of mitochondrial biogenesis by L. donovani metacyclic promastigotes, we compared the responses of BMM infected with unopsonized or serum-opsonized WT, Δ*lpg1*, or Δ*lpg1*+*LPG1* L. donovani metacyclic promastigotes. As shown in Fig. S5A and B, unopsonized and opsonized WT and Δ*lpg1*+*LPG1* L. donovani promastigotes induced a 2-fold increase in the ratio of *MT-16S* relative to *HK2* (mt/n DNA ratio) and a 2- to 4-fold increase in the expression of *PARGC1A* at 24 h postinfection. In contrast, neither unopsonized nor opsonized Δ*lpg1* L. donovani promastigotes stimulated an increase in the mt/n DNA ratio and the expression of *PARGC1A*. Collectively, these results support the notion that LPG is essential but not sufficient for the induction of mitochondrial biogenesis and expression of the respiratory chain components by live L. donovani metacyclic promastigotes.

10.1128/mbio.02578-22.4 FIG S4 (A and B) BMM were infected with either WT, Δ*lpg1*, heat-killed WT, or heat-killed Δ*lpg1* L. donovani metacyclic promastigotes, and at 8 h postinfection (A) the mitochondrial DNA/nuclear DNA ratio (*MT-16S/HK2*) and (B) the kinetics of *PARGC1A* expression were determined. BMM were fed either L. donovani metacyclic promastigotes, zymosan, or LPG-coated zymosan at the indicated time points. (C) The distribution of Tom20 (red) was monitored by confocal microscopy. DAPI staining for DNA is in blue, and 2×-enlarged insets of important regions are shown. (D and E) The mitochondrial DNA/nuclear DNA ratio (*MT-16S/HK2*) (D) and the kinetics of *PARGC1A* expression (E) were determined. BMM were infected with L. donovani metacyclic promastigotes or L. donovani splenic amastigotes isolated from infected hamsters at the indicated time points; (F) the distribution of Tom20 (red) was monitored by confocal microscopy. Blue DAPI staining for DNA and 2×-enlarged insets of representative mitochondria regions are shown. (G) The nuclear DNA/nuclear DNA ratio (*Pecam/HK2*) was determined. The data are presented as mean values ± SEM from three independent experiments. ****, *P* < 0.0001; ***, *P* < 0.001 according to a one-way ANOVA with Dunnett’s multiple-comparison test. Download FIG S4, JPG file, 0.7 MB. Copyright © 2022 Acevedo Ospina et al. 2022 Acevedo Ospina et al. https://creativecommons.org/licenses/by/4.0/ This content is distributed under the terms of the Creative Commons Attribution 4.0 International license.

10.1128/mbio.02578-22.5 FIG S5 (A and B) BMM were infected with opsonized or nonopsonized WT, Δ*lpg1*, or Δ*lpg1*+*LPG1* L. donovani metacyclic promastigotes, and at the indicated time postinfection (A) the mitochondrial DNA/nuclear DNA ratio (*MT-16S/HK2*) and (B) the kinetics of *PARGC1A* expression were determined. (C and D) BMM were infected with either WT or Δ*lpg1* amastigotes isolated *in vitro*, and at the indicated time postinfection (C) the mitochondrial DNA/nuclear DNA ratio (*MT-16S/HK2*) and (D) the kinetics of *PARGC1A* expression were determined. The data are presented as mean values ± SEM from three independent experiments. ****, *P* < 0.0001; ****, *P* < 0.001; **, *P* < 0.01; *, *P* < 0.05 according to a one-way ANOVA with Dunnett’s multiple-comparation test. Download FIG S5, JPG file, 0.9 MB. Copyright © 2022 Acevedo Ospina et al. 2022 Acevedo Ospina et al. https://creativecommons.org/licenses/by/4.0/ This content is distributed under the terms of the Creative Commons Attribution 4.0 International license.

**FIG 3:**
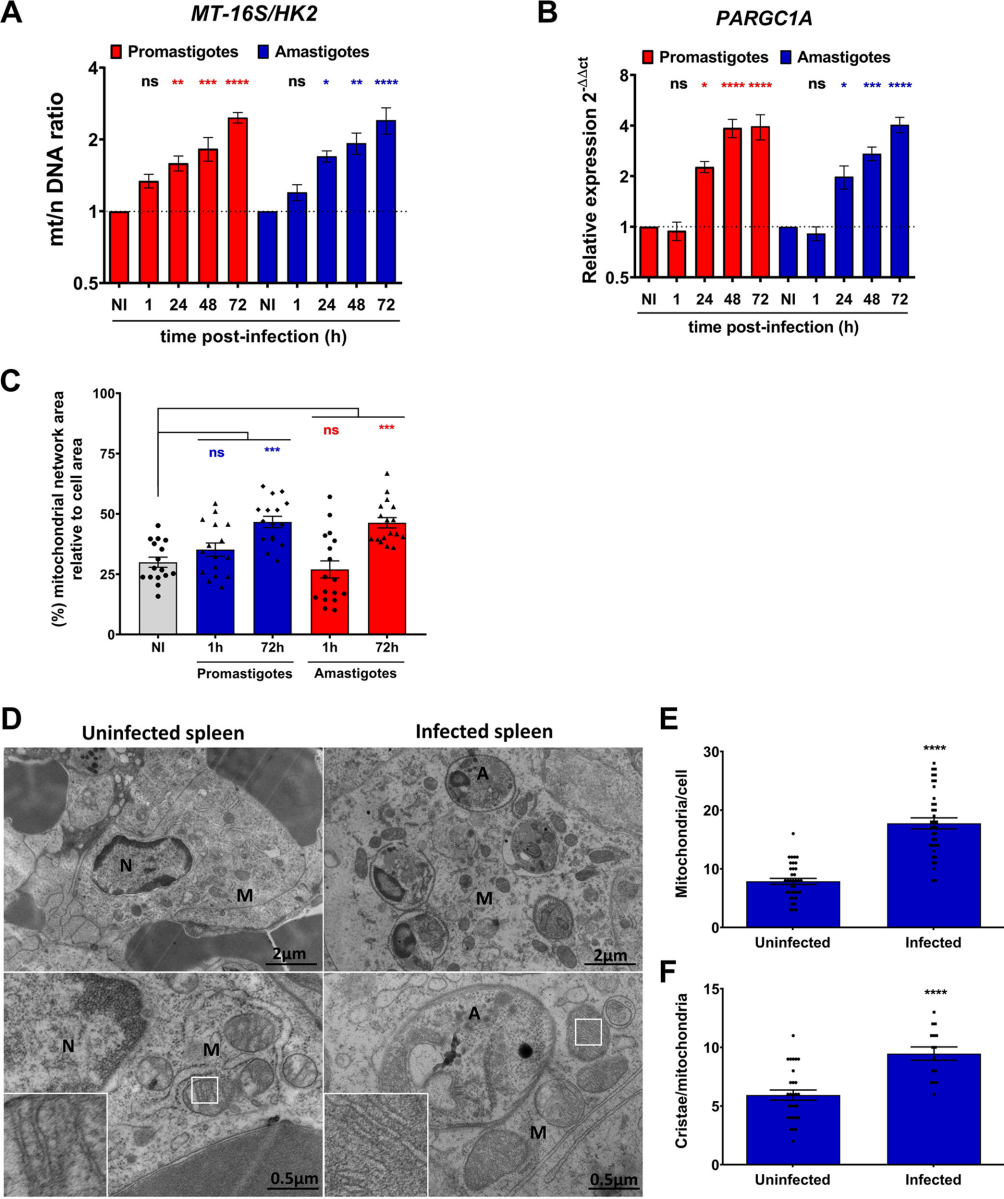
L. donovani amastigotes stimulate host cell mitochondrial biogenesis *in vitro* and *in vivo*. (A to C) BMM were infected with either L. donovani metacyclic promastigotes or amastigotes, and (A) the mitochondrial/nuclear (mt/n) DNA ratio (*MT-16S/HK2*), (B) the kinetics of *PARGC1A* expression, and (C) the mitochondrial network area relative to the cell area were determined. (D) Electron microscopy assessment of spleen from uninfected and L. donovani-infected hamsters. A, M, and N represent amastigotes, mitochondria, and nuclei, respectively. 5×-enlarged insets of representative mitochondrial regions are shown. (E and F) The numbers of mitochondrial (E) and cristae (F) were determined. The data are presented as mean values ± SEM from three independent experiments. (A to C) ****, *P < *0.0001; **, *P < *0.01; *, *P* < 0.05 according to a one-way ANOVA with Dunnett’s multiple-comparation test. (E and F) ****, *P* < 0.0001 according to an unpaired *t* test with Welch’s correction.

Following internalization by macrophages, promastigotes differentiate into amastigotes, which are the mammalian-adapted forms of the parasite that replicate within phagolysosomes (33). To determine whether increased mitochondrial biogenesis induced during the internalization of metacyclic promastigotes is a transient event or persists as the parasites differentiate and replicate, we assessed the mt/n (*MT-16S/HK2*) DNA ratio and the expression of *PARGC1A* up to 72 h following the infection of BMM with L. donovani metacyclic promastigotes. As shown in Fig. 3A and B, compared to uninfected BMM, a 2-fold increase in the mt/n DNA ratio was maintained in infected BMM up to 72 h, whereas expression of *PARGC1A* was increased by 4-fold at 48 h and 72 h postinfection, at the time when promastigotes are fully differentiated into amastigotes. These results led us to assess mitochondrial biogenesis in BMM infected with L. donovani amastigotes isolated from the spleen of infected hamsters. Similar to metacyclic promastigotes, amastigotes induced a 2-fold increase in the mt/n (*MT-16S/HK2*) DNA ratio at 48 h and 72 h postinfection and a 4-fold increase in the expression of *PPARGC1A* at 72 h postinfection compared to uninfected BMM (Fig. 3A and B). These changes in the mt/n (*MT-16S/HK2*) DNA ratio correlated with an increased mitochondrial network area relative to cell area in BMM infected with either promastigotes or amastigotes for 72 h (Fig. 3C, Fig. S4F). As expected, infection with L. donovani metacyclic promastigotes did not change the BMM nuclear/nuclear (*Pecam/HK2*) DNA ratio over the course of infection (Fig. S4G). To further investigate the mechanism by which amastigotes stimulate mitochondrial biogenesis, we used amastigotes recovered from BMM infected for 24 h with either WT or Δ*lpg1* L. donovani metacyclic promastigotes. We infected BMM with either WT or Δ*lpg1* L. donovani amastigotes, and we assessed the mt/n (*MT-16S/HK2*) DNA ratio and the expression of *PARGC1A* at 24 h postinfection. As shown in Fig. S5C and D, similar to splenic amastigotes, WT amastigotes isolated from infected BMM promoted an increase in the mt/n (*MT-16S/HK2*) DNA ratio and *PARGC1A* gene expression compared to uninfected BMM. In contrast, the mt/n (*MT-16S/HK2*) DNA ratio and *PARGC1A* gene expression remained unaltered in BMM infected with Δ*lpg1* amastigotes compared to uninfected BMM (Fig. S5A and B). Collectively, these results suggest that LPG or a structurally related glycolipid contributes to the induction of mitochondrial biogenesis by L. donovani amastigotes. Next, we assessed the impact of L. donovani infection on mitochondria in the spleen of infected hamsters. Using transmission electron microscopy, we quantified the number of mitochondria and the number of cristae per mitochondria in cells from the spleens of uninfected and infected hamsters. As shown in Fig. 3D to F, we observed a 2-fold increase in the number of mitochondria per cell in the spleen of infected hamsters compared to cells from uninfected spleens. Importantly, we observed a significant increase in the number of cristae per mitochondrion in infected spleens compared to uninfected spleens. We also observed that L. donovani-containing vacuoles are surrounded by mitochondria (Fig. 3D), suggesting a repositioning of these organelles in infected cells. Collectively, these results are consistent with the notion that *Leishmania* induces mitochondrial biogenesis in host cells.

### TLRs mediate host cell mitochondrial biogenesis induced by L. donovani metacyclic promastigotes.

**FIG 4:**
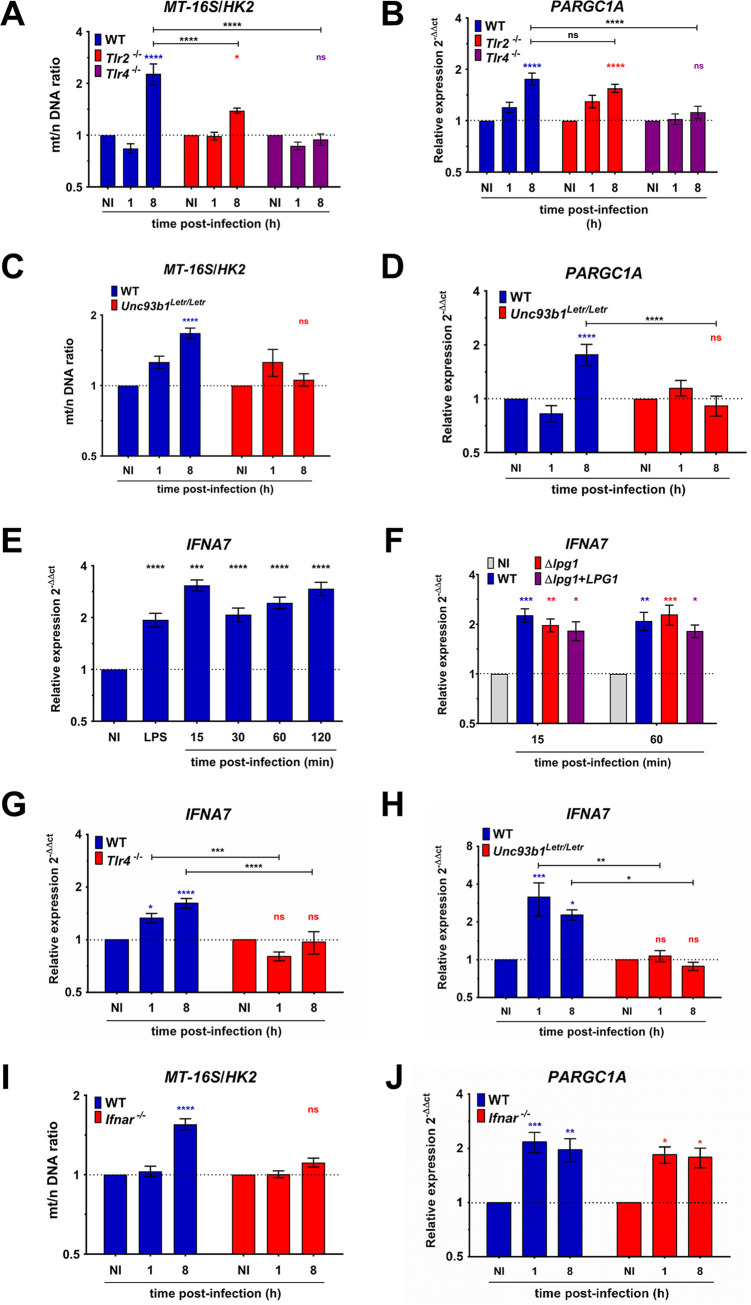
TLRs and the IFN-I signaling axis mediate host cell mitochondrial biogenesis induced by L. donovani metacyclic promastigotes. BMM from either WT, *Tlr2^−/−^*, *Tlr4^−/−^*, or *Unc93b1^Letr/Letr^* mice were infected with WT L. donovani metacyclic promastigotes. (A to D) At the indicated time points, the mt/n DNA ratio (*MT-16S/HK2*) and *PARGC1A* expression were determined. (E) BMM were infected with WT L. donovani metacyclic promastigotes, and at the indicated time points *IFNA7* expression was determined. (F) BMM were infected with either WT, Δ*lpg1*, or Δ*lpg1*+*LPG1* L. donovani metacyclic promastigotes, and at the indicated time points *IFNA7* expression was determined. (G and H) BMM from either WT, *Tlr4^−/−^*, or *Unc93b1^Letr/Letr^* mice were infected with WT L. donovani metacyclic promastigotes, and at the indicated time points *IFNA7* expression was determined. (I and J) BMM from either WT or *Ifnar^−/−^* mice were infected with WT L. donovani metacyclic promastigotes, and at the indicated time points the (I) mt/n DNA ratio (*MT-16S/HK2*) and (J) *PARGC1A* expression were determined. The data are presented as mean values ± SEM from three independent experiments. (A to D and F to J) ****, *P* < 0.0001; ***, *P* < 0.001; **, *P* < 0.01; *, *P* < 0.05 according to a two-way ANOVA with Dunnett’s multiple-comparation test. (E) ****, *P* < 0.0001; ***, *P* < 0.001 according to a one-way ANOVA with Dunnett’s multiple-comparation test.

We previously showed that LPG is shed from the surface of internalized promastigotes and traffics out of the parasitophorous vacuole (34). However, we did not observe significant colocalization between LPG and mitochondria at various time points postinfection (Fig. S6), indicating that LPG acts on mitochondria through receptor-mediated signaling pathways. Several TLRs have been implicated in the recognition of *Leishmania* and *Leishmania*-derived components by various immune cell types and in the modulation of host cell responses to infection (35–39). We therefore sought to assess the contribution of TLRs to the induction of mitochondriogenesis triggered by L. donovani metacyclic promastigotes. Previous studies revealed that depending on the *Leishmania* species and immune cell types involved, LPG may be recognized by either TLR2, TLR4, or both (40–47). To determine whether these receptors are required for the induction of mitochondrial biogenesis, we infected BMM from WT, *Tlr2*^−/−^, and *Tlr4*^−/−^ mice with L. donovani metacyclic promastigotes and at 1 h and 8 h postphagocytosis, we assessed the mt/n (*MT-16S/HK2*) DNA ratio by qPCR. As shown in Fig. 4A, an increase in the mt/n DNA ratio induced by L. donovani metacyclic promastigotes was completely abrogated in the absence of TLR4, whereas a partial increase in the mt/n DNA ratio occurred in the absence of TLR2. Next, we evaluated by real-time quantitative PCR (RT-qPCR) the expression kinetics of the *PARGC1A* gene. As shown in Fig. 4B, induction of *PARGC1A* gene expression by L. donovani metacyclic promastigotes required TLR4, whereas TLR2 was dispensable. We included in our analyses the expression of inducible nitric oxide synthase (iNOS), interleukin-6 (IL-6), and IL-10 using peptidoglycan (PGN) and LPS in BMM from *Tlr2*^−/−^ and *Tlr4*^−/−^ mice as controls for the validation of the TLR-dependent pathways (Fig. S7A and B). These results establish a role for TLR4 in mediating mitochondriogenesis in response to L. donovani metacyclic promastigotes. Endosomal TLRs also contribute to host cell responses triggered by *Leishmania* (48–53). To determine the potential role(s) of endosomal TLRs in mitochondriogenesis, we used BMM derived from *Unc93b1^Letr/Letr^* mice (54). This mutation in the *Unc93b1* gene precludes proper assembly and function of TLR3, TLR7, and TLR9. Expression of IL-6, IL-10, and hypoxia-inducible factor 1α (HIF-1α) is abrogated in BMM from *Unc93b1^Letr/Letr^* stimulated with poly(I·C) but not with LPS (Fig. S7C and D). As shown in Fig. 4C and D, L. donovani metacyclic promastigotes failed to increase the mt/n DNA ratio and *PARGC1A* gene expression in *Unc93b1^Letr/Letr^* BMM, indicating that similar to TLR4, endosomal TLRs are required for the induction of host cell mitochondrial biogenesis by L. donovani promastigotes.

10.1128/mbio.02578-22.6 FIG S6 BMM were infected with L. donovani metacyclic promastigotes at the indicated time points. The mitochondrial network is in red (Tom20), LPG is in green (PG), and DAPI staining for DNA is in blue. 5×-enlarged insets of representative mitochondrial regions are shown. Download FIG S6, JPG file, 0.8 MB. Copyright © 2022 Acevedo Ospina et al. 2022 Acevedo Ospina et al. https://creativecommons.org/licenses/by/4.0/ This content is distributed under the terms of the Creative Commons Attribution 4.0 International license.

10.1128/mbio.02578-22.7 FIG S7 (A to D) BMM from either WT, *Tlr2^−/−^*, *Tlr4^−/−^*, or *Unc93b1^Letr/Letr^* mice were stimulated with (A) 10 μg/mL PGN for 18 h, (B and D) 100 ng/mL LPS for 6 h, or (C) 10 μg/mL poly I:C for 6 h, and transcript levels of NOS, IL-6, IL-10, and HIF-1α were analyzed. (E) BMM were infected with WT L. donovani metacyclic promastigotes, and at the indicated time points the transcript levels for *IFNB1* were analyzed. (F) BMM were infected with either WT, Δ*lpg1*, or Δ*lpg1*+*LPG1* L. donovani metacyclic promastigotes, and at the indicated time points the transcript levels for *IFNB1* were analyzed. (G) BMM were infected with L. donovani metacyclic promastigotes, and at the indicated time points the IFN-α levels were analyzed. (H) BMM from either WT or *Tlr2^−/−^* mice were infected with WT L. donovani metacyclic promastigotes, and at the indicated time points the transcript levels for *IFNA7* were analyzed. The data are presented as mean values ± SEM from three independent experiments. (A to D, F, and G) ****, *P* < 0.0001; **, *P* < 0.01; *, *P* < 0.05 according to a two-way ANOVA with Dunnett’s multiple-comparison test. (E) ****, *P* < 0.0001 according to a one-way ANOVA with Dunnett’s multiple-comparison test. Download FIG S7, JPG file, 0.9 MB. Copyright © 2022 Acevedo Ospina et al. 2022 Acevedo Ospina et al. https://creativecommons.org/licenses/by/4.0/ This content is distributed under the terms of the Creative Commons Attribution 4.0 International license.

### Host cell mitochondrial biogenesis induced by L. donovani metacyclic promastigotes requires the type I IFN signaling pathway.

**FIG 5:**
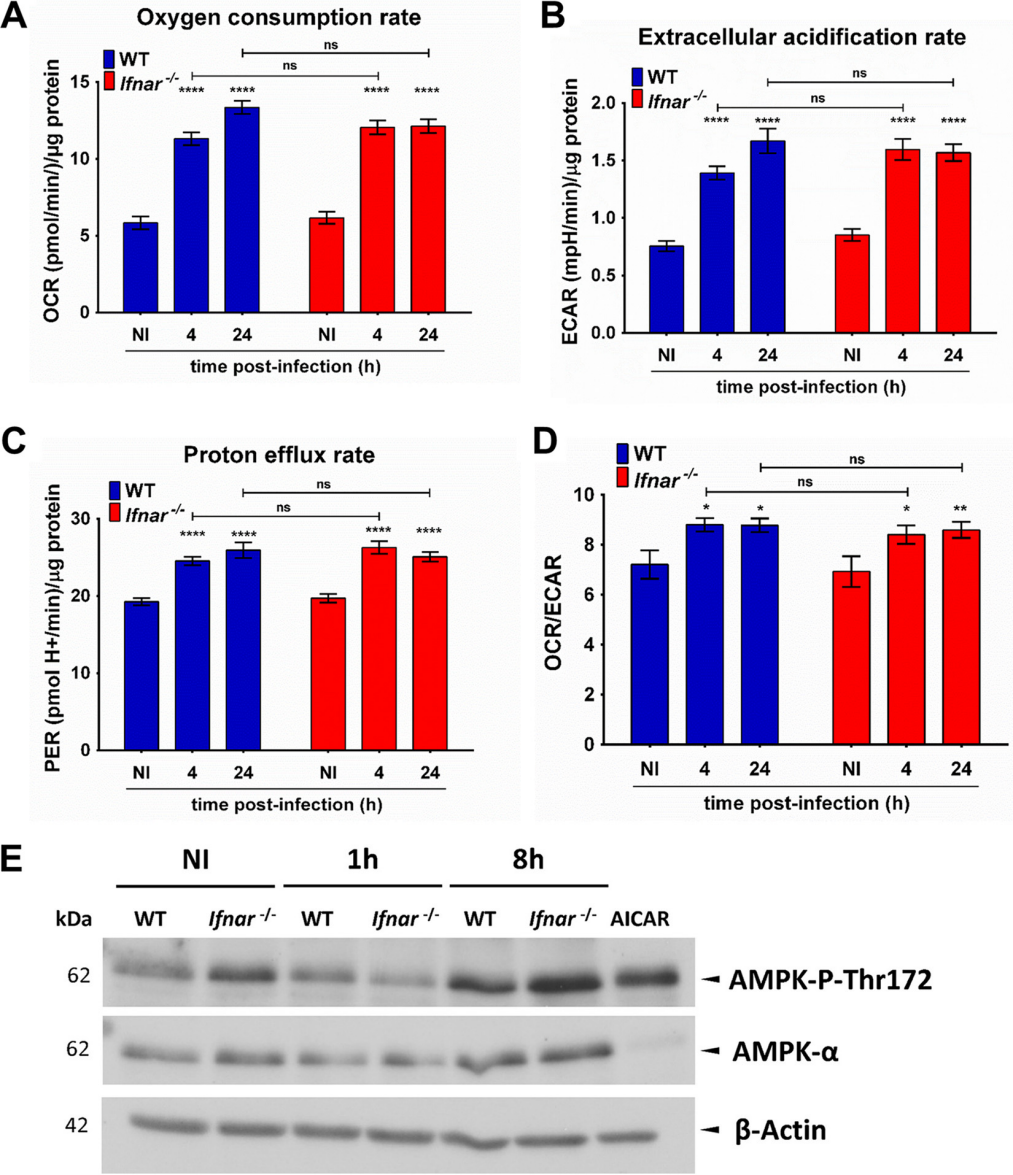
Alteration of host cell bioenergetic metabolism by L. donovani metacyclic promastigotes is independent of IFNAR. (A to D) BMM from either WT or *Ifnar ^−/−^* mice were infected with WT L. donovani metacyclic promastigotes, and at 4 h and 24 h postinfection the OCR, ECAR, and PER and the OCR/ECAR ratio were determined. The readouts in each sample were normalized using the protein concentration, and the measurements were expressed as (OCR, ECAR and PER)/μg protein. (E) BMM from either WT or *Ifnar*^−/−^ mice were infected with WT L. donovani metacyclic promastigotes, and at 1 h and 8 h postinfection the protein levels of AMPK-P-Thr172 and AMPK were determined by Western blot analysis. BMM were treated with 0.1 mM AICAR for 4 h. β-Actin was used as a loading control. Representative immunoblots for two independent experiments. The data are presented as mean values ± SEM from three independent experiments. (A to D) ****, *P* < 0.0001; **, *P* < 0.01; *, *P* < 0.05 according to a two-way ANOVA with Dunnett’s multiple-comparation test.

Activation of TLRs leads to the production of type I IFNs in response to various pathogens, including *Leishmania* (47, 52, 53, 55–57). Given that type I IFNs were reported to modulate mitochondrial metabolism in plasmacytoid dendritic cells (58), we investigated the possibility that autocrine signaling triggered by these cytokines regulates mitochondrial biogenesis in response to L. donovani. Using RT-qPCR, we first obtained evidence that L. donovani metacyclic promastigotes rapidly induced the expression of *IFNA7* (which encodes IFN-α), but not of *IFNB1* (which encodes IFN-β) in BMM (Fig. 4E, Fig. S7E). The observation that WT, Δ*lpg1*, or Δ*lpg1*+*LPG1* L. donovani metacyclic promastigotes induced similar levels of *IFNA7* gene expression indicated that induction of IFN-α expression is LPG independent (Fig. 4F, Fig. S7F). Expression of *IFNA7* was accompanied by an increase in the secretion of IFN-α by BMM infected with L. donovani for 1 h, 8 h, and 24 h compared to uninfected BMM (Fig. S7G). The abolition of *IFNA7* gene expression observed in both *Tlr4^−/−^* and *Unc93b1^Letr/Letr^* BMM (Fig. 4G and H) suggested that TLR4 and endosomal TLRs act in concert to mediate the production of type I IFN in response to L. donovani metacyclic promastigotes. In contrast, the absence of TLR2 had no effect on the induction of *IFNA7* expression by L. donovani promastigotes (Fig. S7H). We next verified whether type I IFN participates in L. donovani-induced mitochondriogenesis, by infecting BMM derived from mice lacking the IFN-I receptor (*Ifnar*^−/−^) (59). In the absence of the IFN-I receptor, the L. donovani-induced increase of the mt/n (*MT-16S/HK2*) DNA ratio was abrogated (Fig. 4I), whereas induction of *PARGC1A* gene expression remained unaffected (Fig. 4J). Since type I IFN signaling contributes to L. donovani-induced mitochondriogenesis, we assessed the contribution of this pathway in the modulation of host macrophage mitochondrial metabolism using live cell extracellular flux analysis. As shown in Fig. 5A to D and Fig. S8A to C, absence of the IFN-I receptor (*Ifnar*^−/−^ BMM) had no effect on the L. donovani-induced changes in the various parameters of mitochondrial metabolism examined (OCR, ECAR, PER, OCR/ECAR, basal respiration, mitochondrial ATP production, maximal respiration), as well as on the phosphorylation of AMPK (Fig. 5E). These results indicate that L. donovani induces an LPG-independent expression of IFN-α, which acts in an autocrine manner to stimulate an increase in the mt/n DNA ratio.

10.1128/mbio.02578-22.8 FIG S8 (A to C) BMM from either WT or *Ifnar ^−/−^* mice were infected with WT L. donovani metacyclic promastigotes, and at the indicated time points, (A) the basal respiration, (B) the mitochondrial ATP production, and (C) the maximal respiration were analyzed. The readouts in each sample were normalized using the protein concentration, and the measurements were expressed as OCR/μg protein. The data are presented as mean values ± SEM from three independent experiments. ****, *P* < 0.0001; ***, *P* < 0.001; **, *P* < 0.01; *, *P* < 0.05 according to a two-way ANOVA with Dunnett’s multiple-comparison test. Download FIG S8, JPG file, 0.7 MB. Copyright © 2022 Acevedo Ospina et al. 2022 Acevedo Ospina et al. https://creativecommons.org/licenses/by/4.0/ This content is distributed under the terms of the Creative Commons Attribution 4.0 International license.

### Mitochondrial biogenesis contributes to the ability of L. donovani promastigotes to colonize BMM.

**FIG 6:**
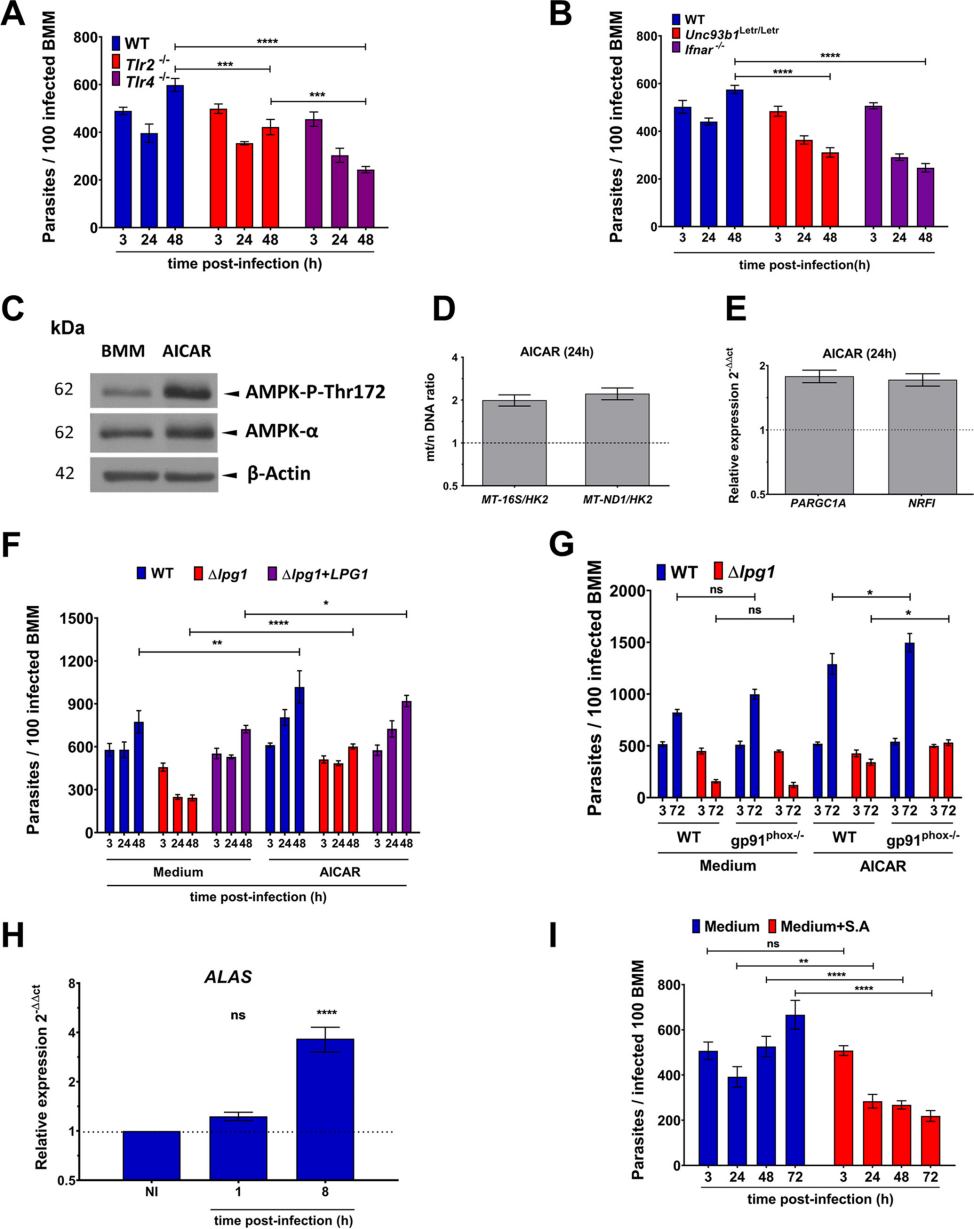
Mitochondrial biogenesis contributes to the ability of L. donovani promastigotes to colonize BMM. (A and B) WT, *Tlr2^−/−^*, *Tlr4^−/−^*, *Unc93b1^Letr/Letr^*, or *Ifnar ^−/−^* BMM were infected with L. donovani metacyclic promastigotes, and parasitemia was assessed at the indicated time points. (C) BMM were treated with 0.1 mM AICAR for 4 h, and the levels of AMPK-P-Thr172 and AMPK were determined by Western blot analysis. β-Actin was used as a loading control. Representative immunoblots for two independent experiments. (D and E) BMM were incubated for 24 h with 0.1 mM AICAR and the (D) mt/n DNA ratio (*MT-16S/HK2* and *MT-ND1/HK2*) and (E) *PARGC1A* and *NRF1* expression were determined. (F) BMM were incubated with 0.1 mM AICAR for 4 h prior to infection with either WT, Δ*lpg1*, or Δ*lpg1+LPG1* L. donovani metacyclic promastigotes. At the indicated time points (with or without AICAR), parasite burden was assessed. (G) WT or gp91*^phox^*^-/-^ BMM were incubated with 0.1 mM AICAR for 4 h prior to infection with either WT or Δ*lpg1* L. donovani metacyclic promastigotes. At the indicated time points (with or without AICAR), parasite burden was assessed. (H) BMM were infected with L. donovani metacyclic promastigotes, and at the indicated time points *ALAS1* expression was assessed. (I) BMM were infected with L. donovani metacyclic promastigotes in the absence or presence 100 μM S.A., and parasite burden was assessed at the indicated time points. The data are presented as means values ± SEM from three independent experiments. (A, E, and F,) ****, *P* < 0.0001; ***, *P* < 0.001; **, *P* < 0.01; *, *P* < 0.05 according to a two-way ANOVA with Dunnett’s multiple-comparation test.

We next investigated the biological relevance of LPG-induced mitochondrial biogenesis with respect to the ability of L. donovani metacyclic promastigotes to colonize macrophages. Since TLR4, endosomal TLRs, and the IFN-I receptor are essential for the induction of mitochondrial biogenesis (Fig. 4A to D and I to J), we compared the fate of WT L. donovani metacyclic promastigotes in wild-type, *Tlr4*^−/−^, *Unc93b1^Letr/Letr^*, and *Ifnar*^−/−^ BMM. Given that TLR2 has no influence on mitochondrial biogenesis, we included *Tlr2*^−/−^ BMM as a control. As shown in Fig. 6A and B, L. donovani metacyclic promastigotes replicated over 48 h postphagocytosis in wild-type BMM. The absence of TLR2 had no impact on the internalization and subsequent survival of L. donovani, although we observed a slight reduction in replication after 48 h in *Tlr2*^−/−^ BMM compared to wild-type BMM. In contrast, survival of L. donovani metacyclic promastigotes and their subsequent replication was significantly impaired in the absence of TLR4, endosomal TLRs, or the IFN-I receptor. These results suggested that induction of mitochondrial biogenesis mediated by those TLRs and the IFN-I receptor contributes to the survival and replication of L. donovani in BMM. Since events downstream of those TLRs and the autocrine signaling triggered by IFN-α other than mitochondrial biogenesis may affect the outcome of L. donovani infection, we determined the fate of L. donovani in BMM in which mitochondriogenesis was pharmacologically induced prior to infection. To this end, we infected BMM pretreated with AICAR (60), an activator of AMPK which stimulates mitochondrial biogenesis as assessed by the increased mt/n DNA ratio (*MT-ND1/HK2* and *MT-16S/HK2*), expression of *PARGC1A* and *NRF1*, and phosphorylation of AMPK (Fig. 6C to E). As shown in Fig. 6F, at 24 h and 48 h postinfection, replication of both WT and Δ*lpg1*+*LPG1* metacyclic promastigotes was significantly increased in AICAR-treated BMM compared to unstimulated BMM. Strikingly, whereas the Δ*lpg1* mutant was cleared in unstimulated BMM, there was a significant increase in the parasite burden (3-fold) of this mutant in AICAR-treated BMM at 48 h postinfection compared to unstimulated BMM (Fig. 6F). A recent report indicated that AICAR significantly decreased the production of reactive oxygen species (ROS) in N-formylmethionyl-leucyl-phenylalanine (fMLF)-stimulated macrophages (61). To determine whether the increased survival of the Δ*lpg1* mutant in AICAR-treated BMM was related to impaired ROS production, we compared the fate of WT and Δ*lpg1* L. donovani metacyclic promastigotes in BMM from WT and gp91*^phox^*^–/–^ mice pretreated or not with AICAR. As shown in Fig. 6G, at 72 h postinfection, the absence of gp91*^phox^*^–/–^ had no significant impact on the fate of both WT and Δ*lpg1* L. donovani promastigotes in control BMM, whereas we observed a slight increase in the parasite burden in gp91*^phox^*^–/–^ BMM pretreated with AICAR for both WT and Δ*lpg1* L. donovani metacyclic promastigotes. These results indicate that increased survival of the Δ*lpg1* mutant in AICAR-treated BMM is not related to an inhibition of ROS production. Moreover, these results suggest that pharmacological induction of mitochondrial biogenesis and stimulation of PGC-1α expression creates a metabolically adapted environment favorable to the replication of L. donovani and that enables the avirulent L. donovani Δ*lpg1* mutant to survive in BMM. These observations led us to explore the possible link between heme synthesis in the context of mitochondrial biogenesis (62) and the ability of *Leishmania* to establish infection. Heme synthesis is associated with increased electron transport chain and with enhanced OXPHOS (63–66). The first biosynthetic step occurs in the mitochondrion and is catalyzed by the 5-aminolevulinic acid synthase (ALAS1), which is the rate-limiting heme biosynthetic enzyme (67). Expression of ALAS1 is tightly regulated and is under the control of NRF-1 and PGC-1α (68). Similar to the induction of these two regulators of gene expression associated with mitochondrial biogenesis and metabolism (Fig. 2D, Fig. S3E), we found that L. donovani metacyclic promastigotes induce high *ALAS1* gene expression, as determined by RT-qPCR (Fig. 6H). These results indicate that L. donovani stimulates heme biosynthesis during host cell infection. To assess the impact of heme biosynthesis on the ability of L. donovani to replicate in BMM, we inhibited the second heme biosynthetic enzyme, aminolevulinic acid dehydratase (ALAD), which catalyzes the conversion of 5-aminolevulinic acid into porphobilinogen. To this end, BMM were incubated with 100 μM succinylacetone 4 h prior to infection, and the inhibitor was maintained for 72 h postinfection. At this concentration, succinylacetone had no effect on the growth of L. donovani promastigotes *in vitro* (data not shown). As shown in Fig. 6I, inhibition of ALAD prevented the replication of L. donovani, indicating that heme biosynthesis during mitochondrial biogenesis contributes to the ability of this parasite to colonize macrophages.

## DISCUSSION

In the present study, we investigated the mechanisms by which L. donovani metacyclic promastigotes alter host cell mitochondrial biology. We describe an essential role for the virulence glycolipid LPG in the stimulation of OXPHOS and in the induction of mitochondrial biogenesis in infected macrophages. Additionally, we demonstrate that mitochondriogenesis requires the action of type I IFN, which is induced independently of LPG. Coupled to the observation that pharmacological induction of mitochondrial biogenesis increases the permissiveness of macrophages to L. donovani, this study supports the notion that mitochondrial biogenesis creates a metabolically adapted environment propitious to the replication of the parasite.

Given the importance of LPG for *Leishmania* promastigotes to colonize their host cells (26–28, 69–72), we sought to determine the potential role of this virulence glycolipid in the modulation of host cell mitochondrial biogenesis and metabolism. As previously reported for L. infantum (9), we found that L. donovani metacyclic promastigotes stimulate OXPHOS, enhance macrophage mitochondrial mass, and induce the expression of PGC-1α and NRF1 as well as respiratory gene expression. Taking advantage of a genetically and structurally defined L. donovani mutant defective in the synthesis of LPG (Δ*lpg1*) and of its complement counterpart (Δ*lpg1*+*LPG1*), we obtained evidence that this molecule is essential for the profound changes in host cell mitochondrial biology induced by L. donovani promastigotes. Thus, in addition to its important role in turning off key host defense processes to protect promastigotes against the microbicidal capacity of phagocytic cells (27, 28, 69–72), our work revealed that modification of host cell metabolism represents an important function for LPG. Similar to metacyclic promastigotes, we observed that splenic amastigotes efficiently stimulate mitochondrial biogenesis in BMM and in the spleen of infected hamsters. Previous reports indicated that splenic L. donovani amastigotes express reduced levels of LPG compared to promastigotes (73, 74) and that they express LPG-like glycolipids which share several chemical and structural properties with promastigote LPG (74). The observation that Δ*lpg1* L. donovani amastigotes failed to trigger increase in the mt/n (*MT-16S/HK2*) DNA ratio and in *PARGC1A* gene expression suggests that the levels of LPG or LPG-like glycolipids expressed by amastigotes are sufficient to stimulate mitochondrial biogenesis. Previous reports identified TLR2 and TLR4 as the receptors responsible for the recognition of LPG from various *Leishmania* species (40, 42, 44, 46, 75–77). Our findings indicate that TLR4 is essential for the stimulation of PGC-1α expression and of mitochondrial biogenesis and are consistent with a role for this receptor in the recognition of LPG. However, LPG by itself is not sufficient to stimulate PGC-1α expression and mitochondrial biogenesis, indicating that additional factors are necessary. Hence, we found that mitochondrial biogenesis requires an LPG-independent pathway linked to the expression of type I IFN. Interestingly, this pathway also involves TLR4, suggesting that this receptor plays a dual role in the control of mitochondrial biogenesis. Although we have not investigated the ligand(s) responsible for TLR4-mediated induction of type I IFN, previous studies evidenced a key role for the neutrophil elastase-TLR4 pathway in this process (47). Using BMM from *Unc93b1^Letr/Letr^* mice (54), we found that in addition to TLR4, induction of mitochondrial biogenesis and of type I IFN expression by L. donovani metacyclic promastigotes requires endosomal TLRs. Several studies have highlighted the contribution of these receptors in the host response to various *Leishmania* species (37–39, 41, 48–52, 78). Recent work revealed that TLR3 is the endosomal TLR that mediates type I IFN expression in response to L. donovani (53), consistent with the defective type I IFN expression in L. donovani-infected *Unc93b1^Letr/Letr^* BMM. Little is known concerning the nature of *Leishmania* ligand(s) recognized by TLR3, with the exception of the double-stranded RNA virus LRV1 (48) present in isolates of various *Leishmania* species (79). It is noteworthy that TLR3 contributes to the recognition of L. donovani promastigotes despite the fact that these parasites do not harbor double-stranded RNA viruses (41, 53). The nature of the L. donovani-derived ligand(s) recognized by TLR3 thus remains to be elucidated. One possibility is that extracellular vesicles containing RNA (80) are released within the parasitophorous vacuoles and activate endosomal TLRs. Alternatively, TLR3 may be activated by RNA released by apoptotic parasites present in metacyclic promastigote populations (81).

In the absence of IFNAR, L. donovani promastigotes fail to induce mitochondrial biogenesis, consistent with the notion that type I IFN acts in an autocrine manner in this process. The fact that L. donovani-induced mitochondrial biogenesis does not take place in *Ifnar^−/−^* BMM despite the induction of PGC-1α expression illustrates the complexity of the pathways involved in this process and highlights the multiple roles of PGC-1α in the modulation of energetic metabolism. Interestingly, our results indicate that type I IFN signaling does not play a significant role in the stimulation of OXPHOS and glycolysis in L. donovani-infected macrophages. This contrasts with the recent findings with Mycobacterium tuberculosis infection, which is characterized by a decrease in both glycolysis and mitochondrial respiration (24). In that study, the authors found that type I IFN is directly responsible for the reduced macrophage energy metabolism during M. tuberculosis infection, suggesting that the effects of type I IFN are pathogen and context specific.

Several studies have highlighted a protective role for type I IFN in leishmaniasis (30, 82, 83). However, accumulating evidence indicates that type I IFN signaling also plays a detrimental role for the host, favoring intracellular parasite replication (47, 52, 53, 56, 83–86). Consistently, targeting type I IFN during anti-*Leishmania* drug treatment was shown to improve Th1 cell-mediated immunity (87). Based on our results, it is tempting to speculate that type I IFN contributes to the ability of *Leishmania* to proliferate within its host through the stimulation of mitochondrial biogenesis. In this regard, our results support the notion that induction of mitochondriogenesis by L. donovani promastigotes is important for the host cell colonization process. Indeed, in agreement with previous reports (47, 53), survival and replication of L. donovani was markedly impaired in TLR4-, endosomal-, and IFNAR-deficient macrophages, in which the parasite fails to induce mitochondrial biogenesis. Additionally, pharmacological induction of mitochondrial biogenesis significantly increased the permissiveness of macrophages to L. donovani replication, suggesting that induction of mitochondrial biogenesis creates a metabolically adapted environment propitious to the replication of the parasite. Similar findings were previously reported for L. infantum-infected BMM (9). This contrasts with several pathogens, including Mycobacterium tuberculosis, Haemophilus parasuis, Staphylococcus aureus, and Plasmodium falciparum, whose survival and replication were impaired by AICAR pretreatment of their host cells (88–90). Strikingly, the avirulent LPG-defective L. donovani Δ*lpg1* mutant, which does not induce mitochondrial biogenesis, survived in BMM pretreated with AICAR. This finding suggests that pharmacological stimulation of mitochondrial biogenesis bypasses the requirement for LPG to create conditions favorable to the parasite’s development within host cells.

Because heme is an essential cofactor for several enzymes of the electron transport chain, its synthesis plays a central role in mitochondrial biology and in OXPHOS complex formation and function (63–66). Hence, the first and rate-limiting step of heme synthesis occurs in mitochondria and is catalyzed by ALAS1 to generate 5-aminolevulinic acid (67). Expression of ALAS1 is tightly regulated and is under the control of PGC-1α (68), which also controls mitochondrial biogenesis and oxidative metabolism (13–15, 17). Our findings that L. donovani promastigotes induce *ALAS1* expression and that pharmacological inhibition of the second heme biosynthetic step impaired the ability of L. donovani to replicate within host macrophages support the importance of this parasite to induce mitochondrial biogenesis and of OXPHOS for its development within mammalian hosts. Additionally, *Leishmania* is a heme auxotroph that must acquire heme or heme precursors from the host to develop intracellularly (91). Activation of heme biosynthesis upon host cell colonization may thus also serve to fulfill the heme requirement of *Leishmania* (92). Future studies will be required to elucidate this issue.

In sum, we provide novel information on the mechanisms leading to mitochondrial biogenesis and metabolic reprograming in macrophages infected with L. donovani. Our results are consistent with the notion of pathogen-specific metabolic rewiring (21), which results from the intricate interplay between complex sets of pathogen molecules and host cell receptors.

## MATERIALS AND METHODS

### Ethics statement.

Animal work was conducted in accordance with protocols 1706-06 and 1706-07, which were approved and defined by the Committee Institutionel de Protection des Animaux of the INRS-Armand-Frappier Santé Biotechnologie. These protocols respect the procedures on animal practice stipulated by the Canadian Council on Animal Care (CCAC).

### Animals and parasites.

C57BL/6 (JAX stock no. 000664), *Tlr2^−/−^* ([93], JAX stock no. 004650), *Tlr4^−/−^* (JAX stock no. 029015), and gp91*^phox^*^–/–^ (JAX stock no. 002365) female and male mice were purchased from The Jackson Laboratories. *Ifnar^−/−^* (59) (kindly provided by Alain Lamarre, Institut National de la Recherche Scientifique) and *Unc93b1^Letr/Letr^* mice (54) (kindly provided by Salman Qureshi, McGill University) were bred and housed at the Institut National de la Recherche Scientifique animal facility under specific-pathogen-free conditions and used at 8 to 12 weeks of age. Female HsdHan:AURA hamsters of 4 to 6 weeks of age were purchased from Harlan Sprague Dawley, Inc. Promastigotes of L. donovani (MHOM/ET/67/Hu3:LV9), L. major NIHS (MHOM/SN/74/Seidman), L. amazonensis LV79 (MPRO/BR/72/M 1841), and L. mexicana (MNYC/BZ/62/M379) were cultured in *Leishmania* medium (M199 medium supplemented with 10% heat-inactivated fetal bovine serum [FBS] [HyClone], 100 μM hypoxanthine, 10 mM HEPES, 5 μM hemin, 3 μM biopterin, 1 μM biotin, penicillin [100 U/mL], and streptomycin [100 μg/mL]) at 26°C. The isogenic L. donovani Δ*lpg1* mutant (71) was cultured in M199 medium supplemented with hygromycin (100 μg/mL), and its complemented counterpart L. donovani Δ*lpg1*+*LPG1* (71) was cultured in M199 medium supplemented with hygromycin (100 μg/mL) and zeocin (100 μg/mL). Amastigotes of L. donovani (MHOM/ET/67/Hu3:LV9) were isolated from the spleens of hamsters infected 8 to 12 weeks earlier with 1.5 × 10^8^ amastigotes by intraperitoneal inoculation (94). To isolate *in vitro* amastigotes, AICAR-pretreated BMM were infected with either WT or Δ*lpg1* L. donovani for 24 h and were lysed for 5 min in Dulbecco’s modified Eagle’s medium (DMEM) supplemented with 0.05% SDS.

### Macrophage culture and infections.

Marrow was extracted from the femurs and tibias of 8- to 12-week-old male and female mice and differentiated for 7 days into bone marrow-derived macrophages (BMM) in Dulbecco’s modified Eagle’s medium with glutamine (DMEM; Thermo Fisher Scientific) containing 10% heat-inactivated fetal bovine serum (FBS; HyClone), 10 mM HEPES, pH 7.4, penicillin (100 IU/mL) and streptomycin (100 μg/mL) and supplemented with 15% (vol/vol) L929 cell-conditioned medium as a source of colony-stimulating factor-1 (CSF-1) in a 37°C incubator with 5% CO_2_. BMM were made quiescent by culturing them in DMEM without CSF-1 for 24 h prior to infection or pharmacological treatments. For infections, metacyclic promastigotes were enriched from late stationary-phase cultures using Ficoll gradients, as previously described (95). Complement opsonization of metacyclic promastigotes, heat-killed metacyclic promastigotes, polystyrene beads, zymosan, and LPG-coated zymosan was performed prior to macrophage internalization through incubation in Hank’s balanced salt solution (HBSS) containing 10% C5-deficient serum from DBA/2 mice for 30 min at 37°C. Adherent BMM were then incubated at 37°C with metacyclic promastigotes or particles, and after 3 h of incubation, noninternalized parasites were removed by washing three times with warm HBSS. Since LPG may reduce the phagocytosis of *Leishmania* promastigotes, we ensured that similar levels of infection were achieved by infecting BMM at multiplicities of infection (MOIs) of 7:1 for WT parasites, 6:1 for the Δ*lpg1* mutant, and 8:1 for the Δ*lpg1*+*LPG1*. A particle-to-cell ratio of 7:1 was used for heat-killed parasites, polystyrene beads, and zymosan or LPG-coated zymosan per BMM unless otherwise specified. For infection or treatments shorter than 3 h, noninternalized parasites or particles were removed by washing three times with warm HBSS at the specified time postphagocytosis and immediately processed. Intracellular parasitemia was assessed at the indicated time point by counting the number of parasites per 100 infected BMM upon staining with the Hema 3 staining kit. For pharmacological treatments, BMM were incubated with the following compounds for the indicated time points: 100 ng/mL LPS (Escherichia coli, strain 0127: B8, Sigma) for 6 h, 0.1 mM 5-aminoimidazole-4-carboxamide-1-β-d-ribofuranoside (AICAR, Sigma) for 4 h and 24 h, 10 μg/mL PGN (BioChemika) for 18 h, 10 μg/mL poly(I·C) (Sigma) for 6 h, and 100 μM succinyl acetone (S.A., Sigma) for 4 h.

### Confocal immunofluorescence microscopy.

BMM were seeded in 24-well plates containing microscope coverslips (Fisher Scientific) and infected with either WT, Δ*lpg1*, or Δ*lpg1*+*LPG1* L. donovani metacyclic promastigotes or were fed zymosan and LPG-coated zymosan and amastigotes for the indicated time points. Cells were washed with phosphate-buffered saline (PBS), fixed with 3.7% paraformaldehyde (PFA) for 30 min, and then permeabilized in 0.1% Triton X-100 for 5 min. Then, the samples were blocked in 10% bovine serum albumin for 1 h. Cells were incubated for 1 h using an anti-Tom20 rabbit polyclonal antibody (1:200) and mouse monoclonal antibody (CA7AE, Cedarlane). BMM were next incubated with an appropriate combination of secondary antibodies (anti-rabbit Alexa Fluor 568, 1:500, and anti-IGM Alexa Fluor 488, 1:500) for 1 h. Macrophage and promastigote nuclei were stained with DAPI (Molecular Probes). Coverslips were washed three times with PBS after every step, and all steps were performed at room temperature. Analyses of Tom20 distribution were performed on a LSM780 confocal microscope (Carl Zeiss Microimaging) using Plan Apochromat ×63 oil-immersion differential interference contrast (DIC) (NA 1.64) objective, and images were acquired in sequential scanning mode. Images were processed with ZEN 2012 software. At least 30 cells per condition were analyzed using Icy image analysis software, and statistical differences were evaluated using one-way analysis of variance (ANOVA) followed by Tukey’s posttests (three groups). Data were considered statistically significant when *P* was <0.05, and graphs were plotted with GraphPad Prism 5.

### Quantitative PCR analysis.

Total RNA from BMM was isolated using an RNeasy minikit (Qiagen) according to the manufacturer’s protocol. RNA (2 μg) was reverse transcribed using Oligo(dT) 12-18 primer (Invitrogen), and real-time quantitative PCR (RT-qPCR) experiments were performed in independent biological replicates (at least 3 replicates); reactions were run in at least duplicate for each sample using iTaq Universal SYBR green supermix (Bio-Rad) on a Stratagene mx3005p real-time PCR system using 10 ng cDNA. Gene expression changes were analyzed using the comparative threshold cycle (CT) method (ΔΔ*CT*) (96). Relative mRNA amounts were normalized to the Rps29 gene and expressed as the fold increase compared to noninfected controls. To determine the mitochondrial/nuclear (mt/n) DNA ratio, total BMM DNA was extracted using a DNeasy blood and tissue kit. Quantitative PCR (qPCR) experiments were performed in independent biological replicates (at least 3 replicates), and reactions were run at least in duplicates for each sample using iTaq Universal SYBR green supermix (Bio-Rad) on a Stratagene mx3005p real-time PCR system using 10 ng DNA. The amount of mtDNA present per nuclear genome was determined using the comparative CT method (ΔΔ*CT*) (96). Relative mtDNA amounts were normalized to the hexokinase gene and expressed as the fold increase compared to noninfected controls. The DNA and RNA concentrations were determined by optical density at 260 nm (OD_260_) measurement using a NanoDrop spectrophotometer. The complete list of primers used is shown in Table S1.

10.1128/mbio.02578-22.9 TABLE S1 Oligonucleotides used in this study for RT-qPCR and qPCR. Download Table S1, TIF file, 1.1 MB. Copyright © 2022 Acevedo Ospina et al. 2022 Acevedo Ospina et al. https://creativecommons.org/licenses/by/4.0/ This content is distributed under the terms of the Creative Commons Attribution 4.0 International license.

### Western blot analysis.

Prior to lysis, adherent BMM were placed on ice and washed 3 times with PBS containing 1 mM sodium orthovanadate and 5 mM 1,10-phenanthroline (Sigma). Cells were scraped in the presence of lysis buffer containing 1% NP-40, 50 mM Tris-HCl (pH 7.5), 150 mM NaCl, 1 mM EDTA (pH 8), 10 mM 1,10-phenanthroline, and phosphatase and protease inhibitors (Roche). After 24 h of incubation at −70°C, lysates were centrifuged for 30 min, and the soluble phase was collected. After protein quantification, 20 μg of protein was boiled (100°C) for 5 min in SDS sample buffer and migrated in SDS-PAGE gels. Proteins were transferred onto Hybond-ECL membranes (Amersham Biosciences), blocked for 1 h in Tris-buffered saline (TBS) 1×-0.1% Tween containing 5% BSA, and incubated with primary antibodies (diluted in TBS 1×-0.1% Tween containing 5% BSA) overnight at 4°C and then with suitable horseradish peroxidase (HRP)-conjugated secondary antibodies for 1 h at room temperature. Membranes were incubated in ECL (GE Healthcare), and immunodetection was achieved via chemiluminescence. Densitometric analysis of Western blot bands was done using ImageJ software. Primary antibodies were directed against total AMPKα (23A3, Cell Signaling), AMPKα phosphorylated at Thr172 (2531, Cell Signaling), Tom20 (Abcam), NDUFA 9 (Abcam), antiphosphoglycan (Galβ1,4Manα1-PO_4_) mouse monoclonal antibody (CA7AE, Cedarlane), anti-L. donovani aldolase rabbit polyclonal antibody (kind gift from A. Jardim), and β-actin (Cell Signaling).

### ELISA.

IFN-α levels in supernatants were measured using an enzyme-linked immunosorbent assay (ELISA) according to the manufacturer’s protocol. Each treatment was tested in duplicate. A mouse IFN-α SimpleStep ELISA kit (catalog [cat.] no. ab252352) was purchased from Abcam (Burlingame, CA, USA).

### Electron microscopy.

Uninfected and L. donovani-infected spleens were recovered, and 2-mm^2^ samples were fixed with 1.3% (wt/vol) osmium tetroxide in collidine buffer for 1 h. The dehydration process was done in 25%, 50%, 75%, and 95% solutions of acetone/ethanol in water for 30 min each, followed by two changes of pure acetone/ethanol for 30 min each. Then, the samples were immersed for 16 to 18 h in Spurr:acetone (1:1). After that, the samples were immersed in two successive baths of Spurr mixtures for 2 h each. The samples were cut into smaller pieces, placed in BEEM capsules, filled using SPURR mixtures, and left to stand at room temperature for 18 h. Then, the filled capsules were placed at 60° to 65°C for 30 h to polymerize the resin. The polymerized resins were cut into ultrathin sections on an ultramicrotome and put onto a Formvar- and carbon-covered copper 200-mesh grid. The samples were stained with uranyl acetate in 50% ethanol for 15 min, followed by lead citrate for 5 min. Image acquisitions were made using an electronic microscope (Hitachi H-7100) with an AMT camera.

### Metabolism assays.

The bioenergetic profile of L. donovani-infected BMM was analyzed using an XF-96 extracellular flux analyzer (Seahorse Bioscience). The oxygen consumption rate (OCR), extracellular acidification rate (ECAR), and proton efflux rate (PER) were determined at the indicated time postinfection. BMM were seeded at 8 × 10^4^ cells/well in 100 μL of DMEM in XF-96 cell culture plates, and after an overnight incubation period, cells were infected with WT, Δ*lpg1*, and Δ*lpg1*+*LPG1* L. donovani metacyclic promastigotes. One hour before the defined times of infection, the cells were washed and the medium was changed to XF medium (buffered DMEM supplemented with 4.5 g/L glucose, 2% FBS, 2 mM l-glutamine, 100 U/mL penicillin, and 100 mg/mL streptomycin). The real-time measurement of the bioenergetic profile was obtained under basal conditions and in response to oligomycin (1 μM), fluoro-carbonyl cyanide phenylhydrazone (FCCP, 2 μM), rotenone (1 μM), and antimycin A (1 μM). The nonmitochondrial respiration was obtained by subtracting the rotenone/antimycin A values. The spare respiratory capacity (SRC) was obtained by subtracting FCCP from basal OCR values, and the glycolytic capacity was defined as the variation between oligomycin and basal ECAR values. The procedure used in the experiments was established according to the manufacturer’s (Seahorse) instructions. Glucose dependence was assessed using 2-deoxyglucose (2-DG, 50 mM), mitochondrial β-oxidation dependence was assessed using etomoxir (4 μM), glutaminolysis dependence was assessed using BPTES (3 μM), and peroxisomal β-oxidation dependence was assessed using thioridazine (1 μM). In all cases, the treatments were done 30 min prior the readouts of the specific time point.

### Statistics and reproducibility.

GraphPad Prism 6 software was used to generate the graphs and statistical analyses. All experiments were conducted three independent times, unless otherwise specified in the figure legends. Methods for statistical tests, exact value of *n*, and definition of error bars are indicated in the figure legends; *, *P* < 0.05; **, *P* < 0.01; ***, *P* < 0.001; ****, *P* < 0.0001. All immunoblots and images shown are representative of these independent experiments.

### Data availability.

All data generated or analyzed during this study are included in this published article and its supplemental information files.
