# Supplementary figures and images for: Macrophage Mitochondrial Biogenesis and Metabolic Reprogramming Induced by Leishmania donovani Require Lipophosphoglycan and Type I Interferon Signaling
Source: mBio. 2022 Oct 12;13(6):e02578-22. doi: 10.1128/mbio.02578-22 (PMC9764995; doi:10.1128/mbio.02578-22)

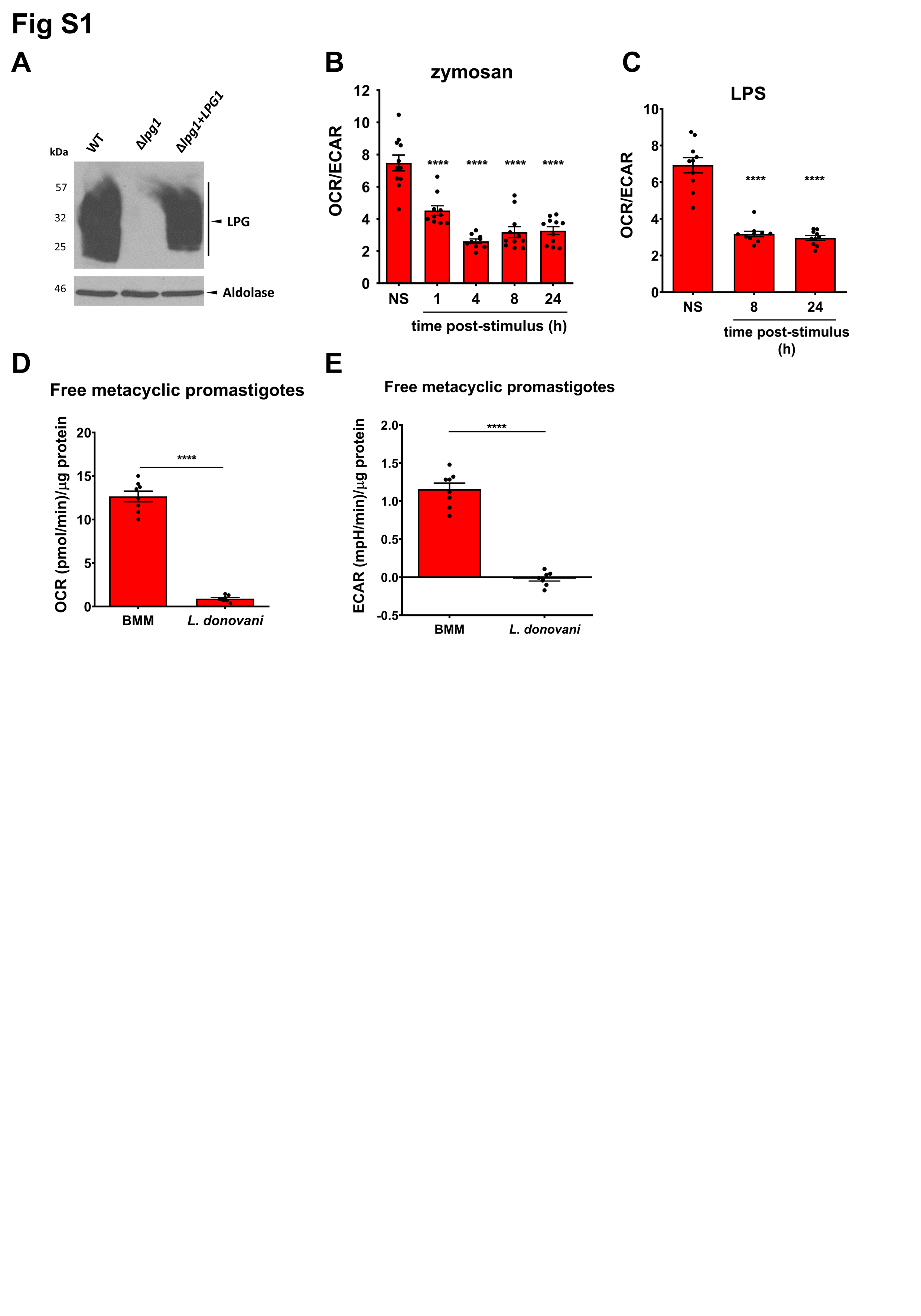

Supplement: FIG S1 [file mbio.02578-22-s0001.tif]

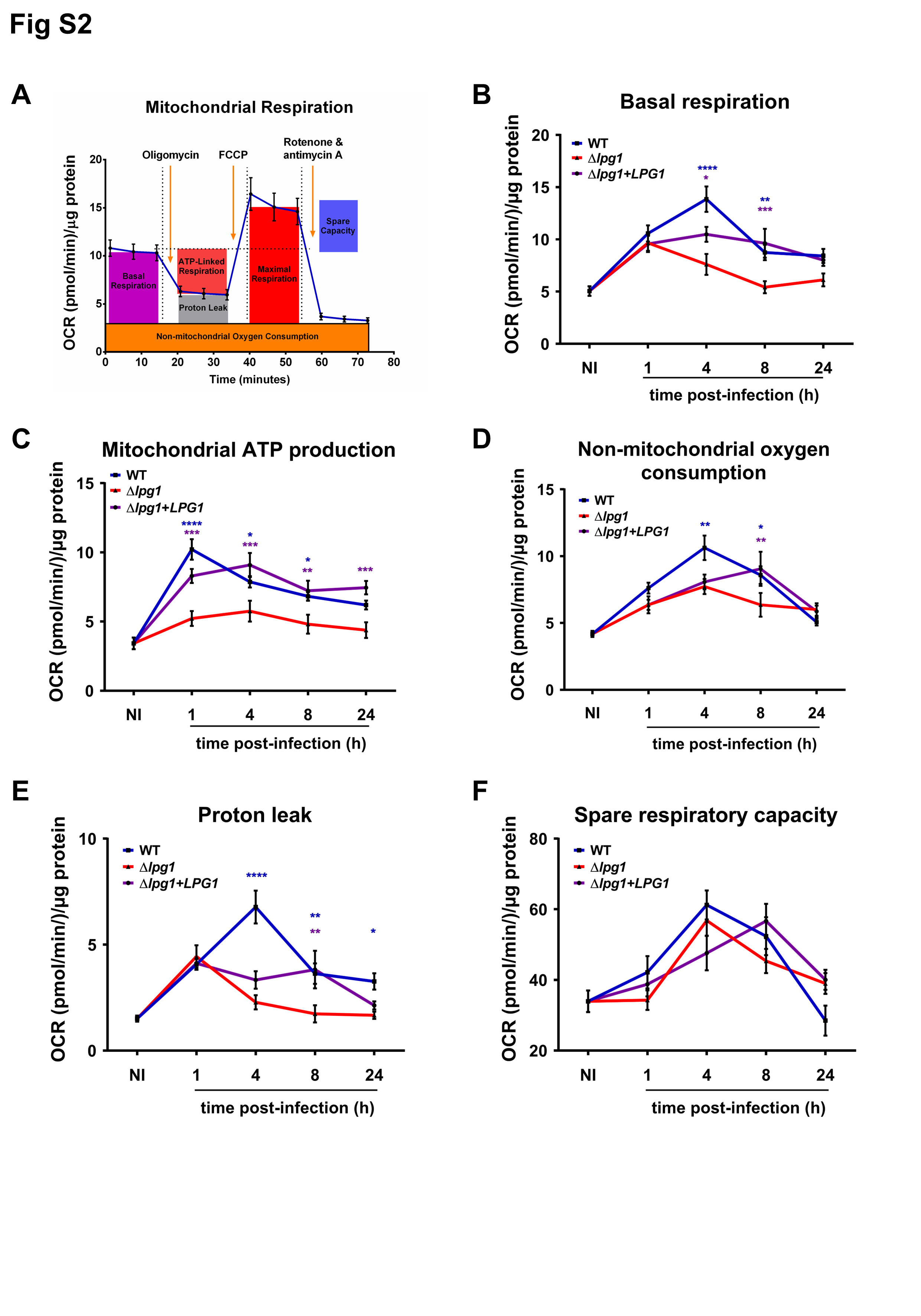

Supplement: FIG S2 [file mbio.02578-22-s0002.tif]

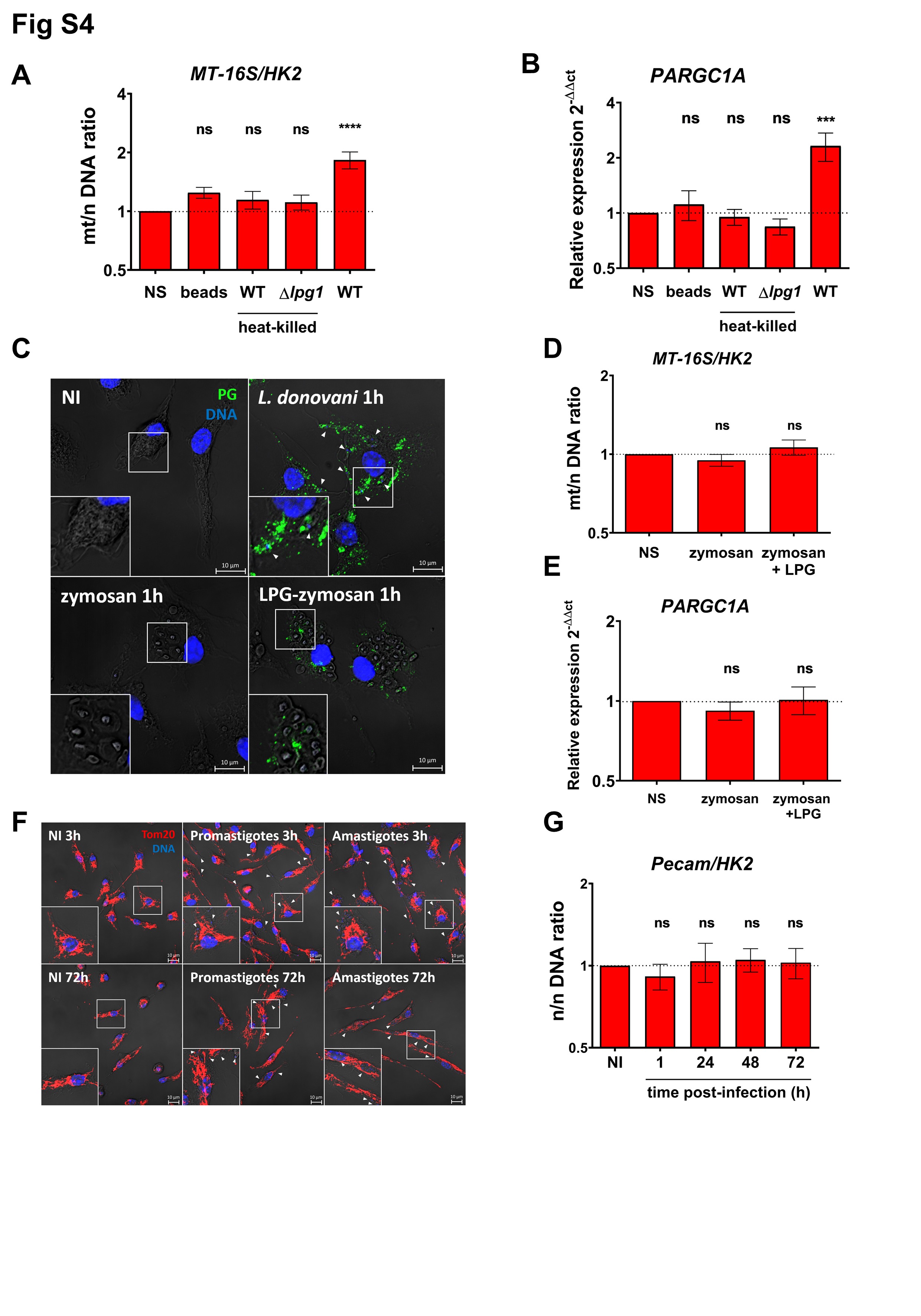

Supplement: FIG S4 [file mbio.02578-22-s0004.jpg]

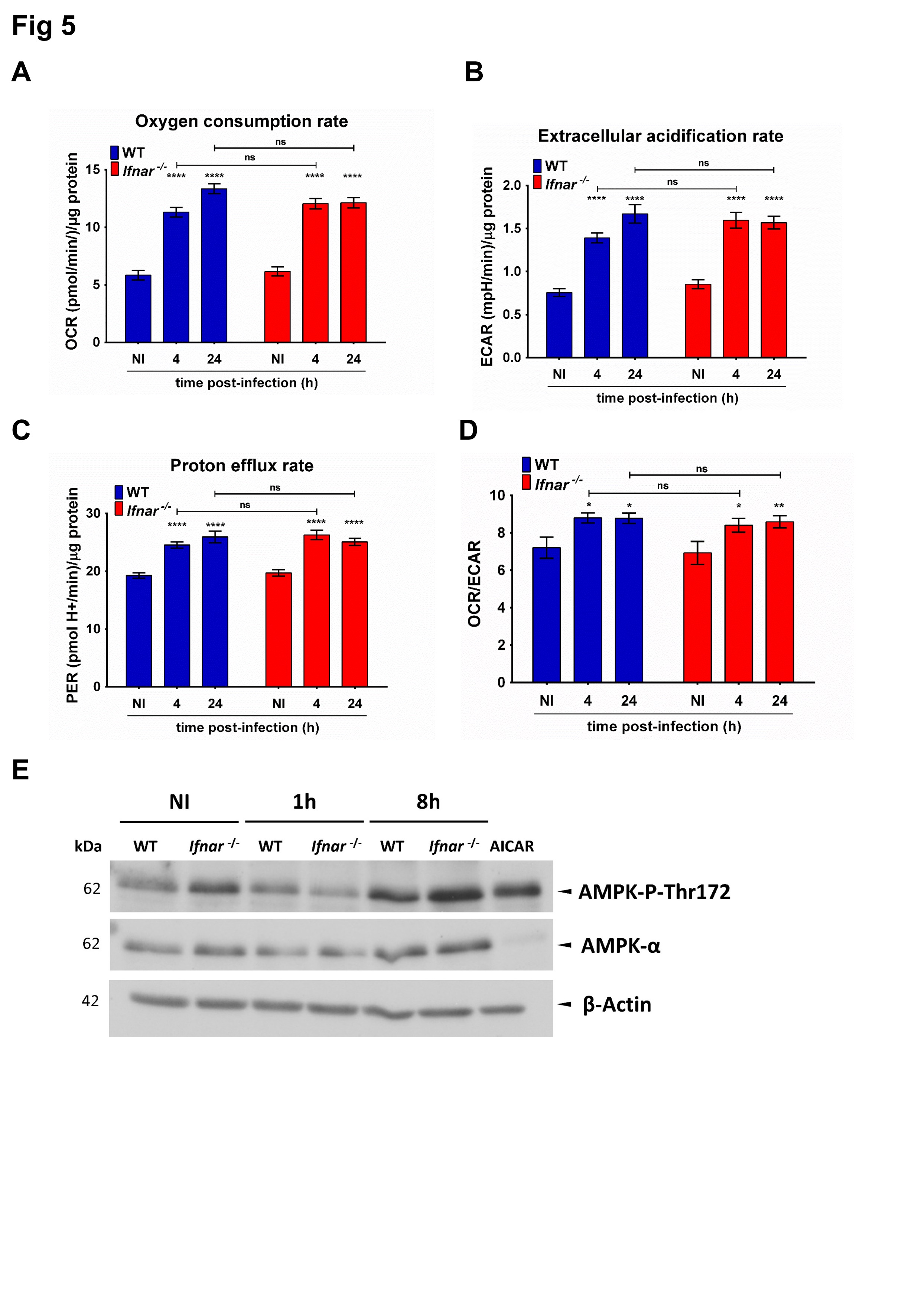

Supplement: FIG S5 [file mbio.02578-22-s0005.jpg]

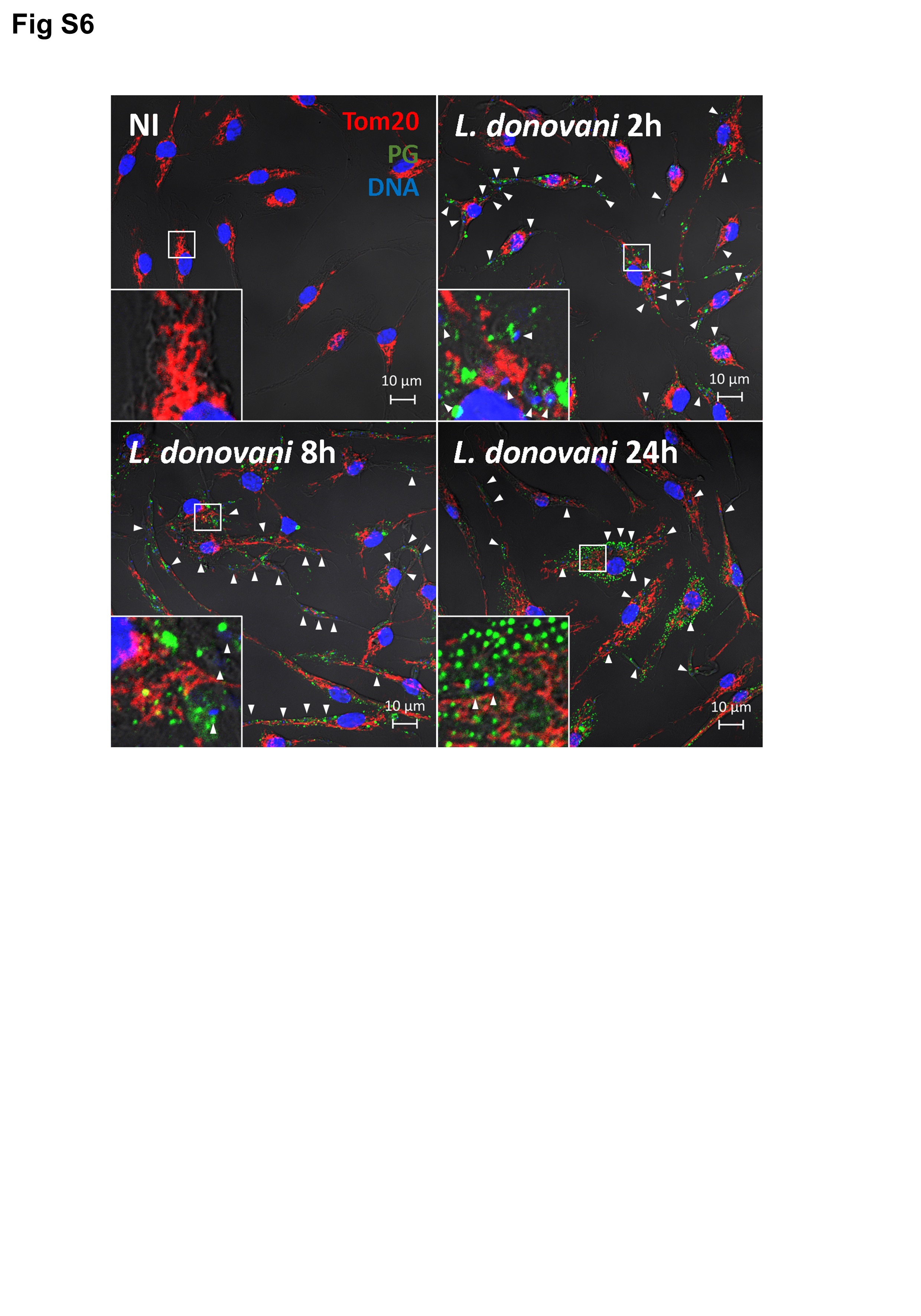

Supplement: FIG S6 [file mbio.02578-22-s0006.jpg]

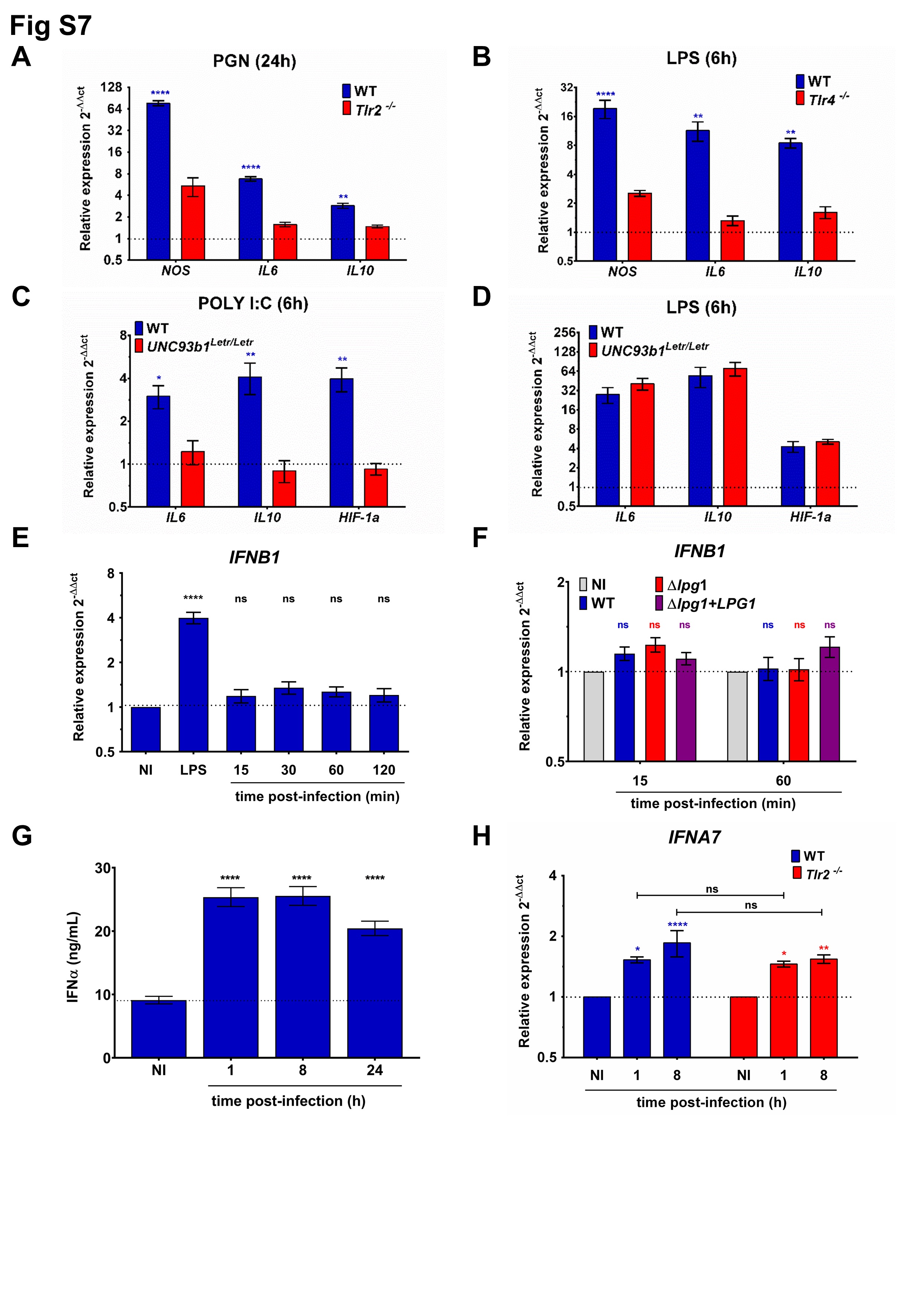

Supplement: FIG S7 [file mbio.02578-22-s0007.jpg]

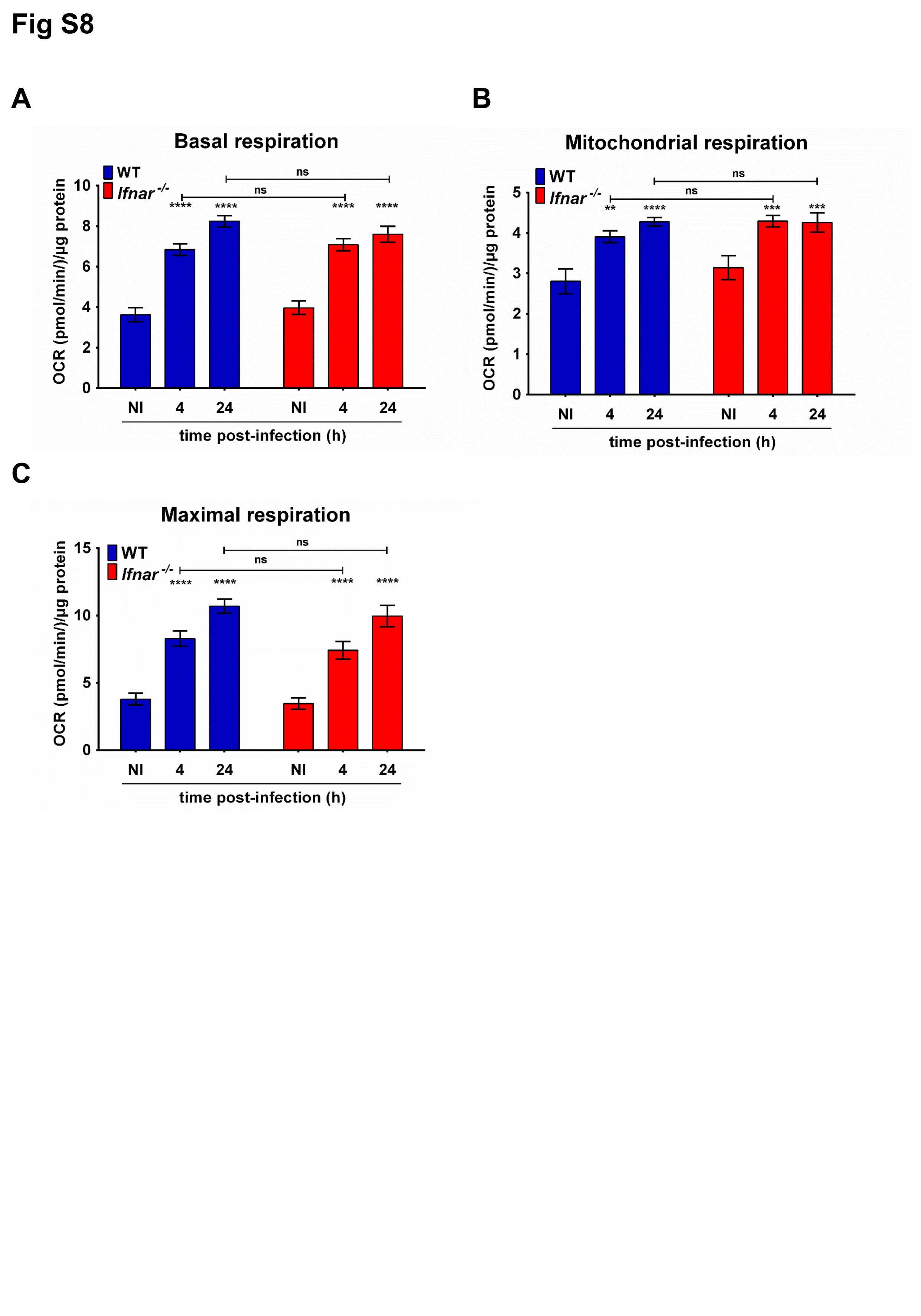

Supplement: FIG S8 [file mbio.02578-22-s0008.jpg]

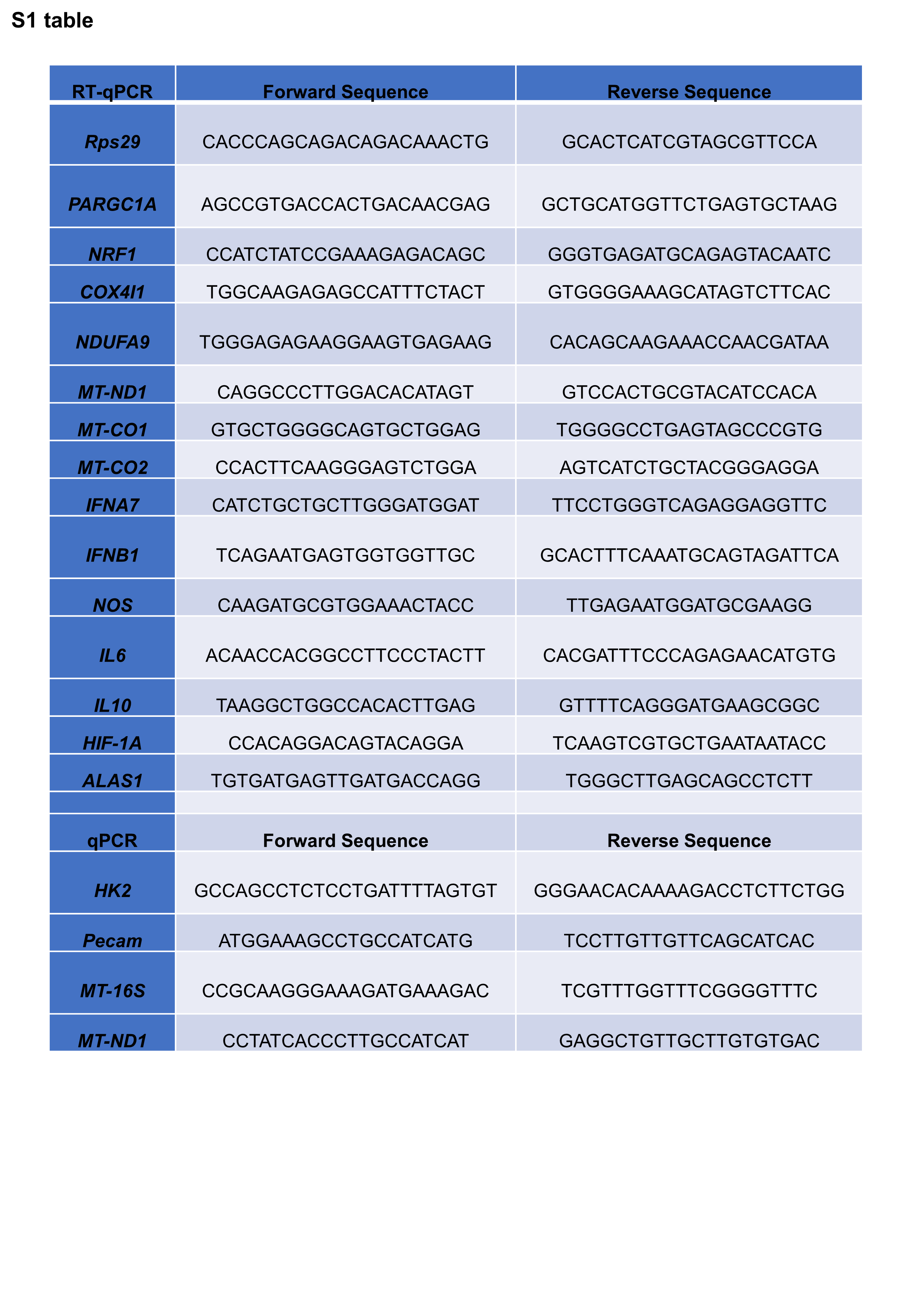

Supplement: TABLE S1 [file mbio.02578-22-s0009.tif]
